# Novel endoscopic optical diagnostic technologies in medical trial research: recent advancements and future prospects

**DOI:** 10.1186/s12938-020-00845-5

**Published:** 2021-01-06

**Authors:** Zhongyu He, Peng Wang, Xuesong Ye

**Affiliations:** 1 Biosensor National Special Laboratory, College of Biomedical Engineering and Instrument Science, Zhejiang University, Hangzhou, 310027 People’s Republic of China; 2 State Key Laboratory of CAD and CG, Zhejiang University, Hangzhou, 310058 People’s Republic of China

**Keywords:** Endoscopy, Optical diagnostic technologies, Endomicroscopy, Biomedical spectroscopy

## Abstract

Novel endoscopic biophotonic diagnostic technologies have the potential to non-invasively detect the interior of a hollow organ or cavity of the human body with subcellular resolution or to obtain biochemical information about tissue in real time. With the capability to visualize or analyze the diagnostic target in vivo, these techniques gradually developed as potential candidates to challenge histopathology which remains the gold standard for diagnosis. Consequently, many innovative endoscopic diagnostic techniques have succeeded in detection, characterization, and confirmation: the three critical steps for routine endoscopic diagnosis. In this review, we mainly summarize researches on emerging endoscopic optical diagnostic techniques, with emphasis on recent advances. We also introduce the fundamental principles and the development of those techniques and compare their characteristics. Especially, we shed light on the merit of novel endoscopic imaging technologies in medical research. For example, hyperspectral imaging and Raman spectroscopy provide direct molecular information, while optical coherence tomography and multi-photo endomicroscopy offer a more extensive detection range and excellent spatial–temporal resolution. Furthermore, we summarize the unexplored application fields of these endoscopic optical techniques in major hospital departments for biomedical researchers. Finally, we provide a brief overview of the future perspectives, as well as bottlenecks of those endoscopic optical diagnostic technologies. We believe all these efforts will enrich the diagnostic toolbox for endoscopists, enhance diagnostic efficiency, and reduce the rate of missed diagnosis and misdiagnosis.

## Introduction

An endoscope is used in medicine to examine the interior of a hollow organ or cavity of the human body [1, 2]. Developments in optical imaging technology continue to promote the revolution of endoscopy. Currently, electronic chromoendoscopic techniques such as narrow-band imaging (NBI, Olympus, Japan) [3–5], linked color imaging (LCI, Fujifilm, Japan) [6–8] and i-scan (Pentax, Japan) [9–11] provide wide-field high-contrast video images to help endoscopists find mucosal abnormalities especially precancers [12]. However, comparing with traditional histopathologic biopsy, electronic chromoendoscopy lacks the capacity to provide more detailed morphologic information about tissue and cell for accurately confirming or staging cancer [13]. Furthermore, although existing commercial endomicroscopy including endocytoscopy (EC, Olympus, Japan) [14] or probe-based confocal laser endomicroscopy (PCLE, Mauna KeaTech, France) [15] can image at cellular information, their detection range is limited to the surface of the mucosa (< 1 mm in depth) which may cause the missed diagnosis of the hidden lesions in deep tissue [16].

Therefore, many studies have been conducted in numerous research centers on novel endoscopic diagnostic technologies to provide more accurate diagnostic information. The advent of these technologies in medical trial research, such as photoacoustic endoscopy (PAE), Raman spectroscopy (RS), two-photon excited fluorescence (TPEF) imaging has opened a new era and created tremendous opportunities for the enhanced identification and biochemical characterization of diseases. These modalities also have the potential to allow non-invasive in vivo “optical biopsy” which differentiates areas of similar clinical characteristics, hence challenging the ex vivo histology which is the only way for definitive cancer diagnosis [17]. In addition, these endoscopic techniques own unprecedented temporal-spatial resolution of imaging with innovative mechanisms such as photoacoustics, optical coherent tomography, and multi-photo effect. By utilizing these novel tissue–photon interacting mechanisms, these techniques can guide biopsies by accessing the subtle mucosal/submucosal abnormalities with higher contrast, better resolution, and penetration depth. Hence, they cut down the number of biopsy times, costs, and risks for patients.

However, most of these technologies are not mature, so they have been neither officially launched into the market nor put into clinical use in the hospital so far. Thus, well-organized large-scale animal experiments and human clinical trials should be executed to further validate and standardize these techniques before approval of the clinical application.

For this review, the PubMed and Springer Link literature search was systematically performed for studies published from 1983 to 2019 about emerging endoscopic imaging and sensing technologies by using the search terms “Endoscopy,” “Endomicroscopy,” “Endoscopic medical imaging,” “Image-enhanced endoscopy,” “Hyperspectral imaging,” “Fluorescence lifetime imaging,” “Photoacoustic endoscopy,” “Optical coherent tomography,” “Coherent anti-Stokes Raman scattering,” “Multi-photon imaging,” “Diffuse reflectance spectroscopy,” “Light-scattering spectroscopy,” “Angle-resolved low coherence interferometry,” “Raman spectroscopy,” “Super-resolution microscopies” and “In vivo clinical trial research.” Further articles were obtained through the review of the quoted references from the selected reference articles. Only full manuscripts and case reports published in English were collected. We included studies that firstly proposed novel modalities of in vivo endoscopic optical detection or that significantly improved technical performances (such as spatiotemporal resolution, real-time performance, SNR, image contrast, etc.) of existing technologies in the last decade. Additionally, we specially presented some technologies that have been tested in human clinical trials in various medical specialties. However, for new or emerging technology where related pieces of literature are scarce, articles with other study designs were also included. This review intents to provide an accurate and comprehensive synopsis, illustrated by a few examples with emphasis on their fundamental principles and technical parameters, of where and how the novel endoscopic optical diagnostic technologies in medical trial research have already contributed to, or probably will contribute to the way of endoscopic detection and treatment. Thus, this article could serve as a guideline for future researchers and promote cooperation between researchers in different disciplines to accelerate the development and clinical application of endoscopic technologies.

## Classifications on novel technologies in medical trial research

**Fig. 1:**
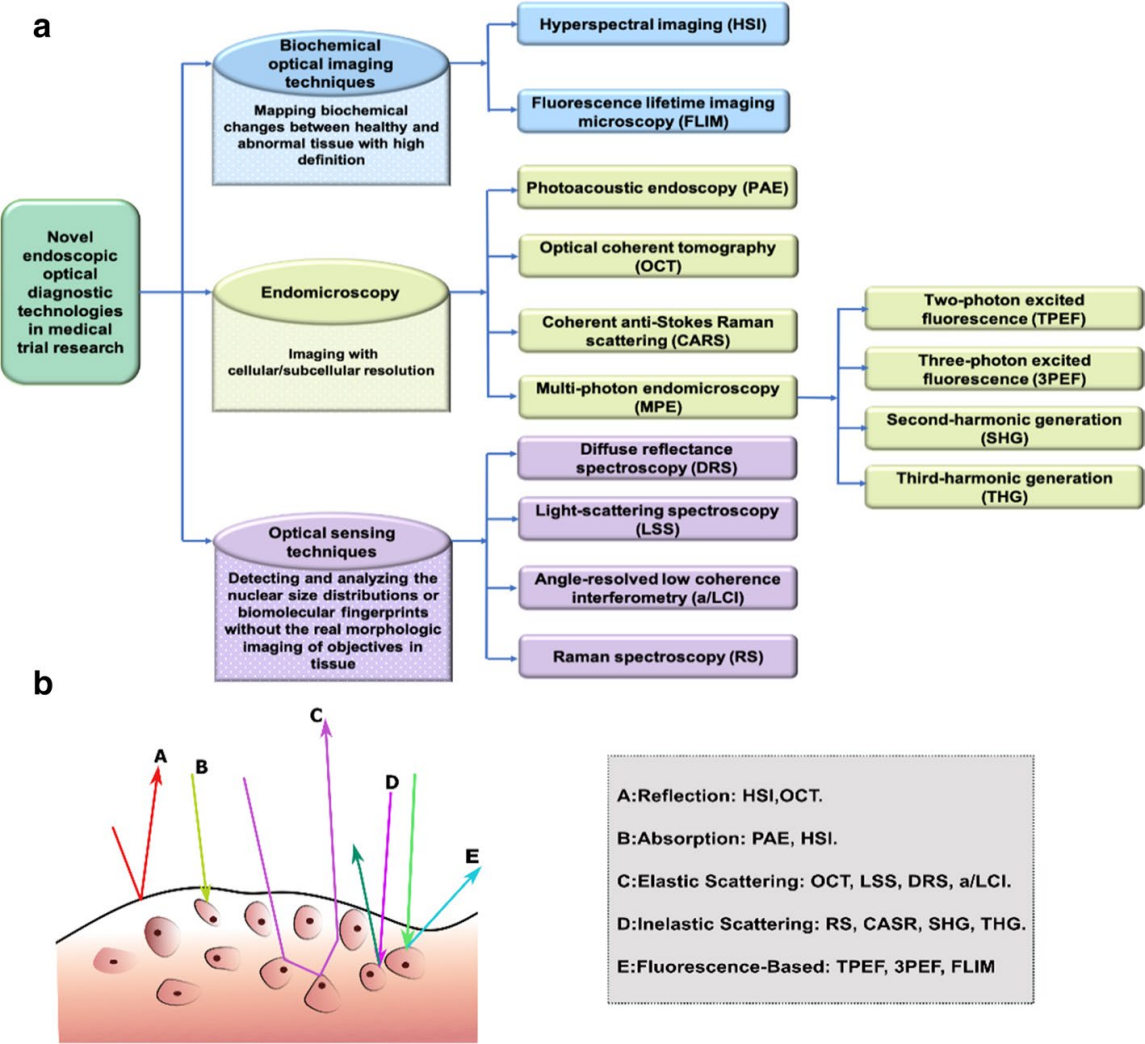
**a** An overview of novel endoscopic optical diagnostic technologies in medical trial research in this review. **b** Optical mechanisms of potential endoscopic biophotonic diagnostic technologies. There is more than one way in how light interacts tissue happens in some techniques, such as HSI and OCT

We divide novel endoscopic optical diagnostic technologies in medical trial research into three categories: endoscopic biochemical optical imaging techniques, endomicroscopy, and optical sensing techniques (Fig. 1a). Label-free biochemical imaging technologies are mainly about mapping biochemical changes between healthy and abnormal tissue with high definition, while endomicroscopy reaches cellular/subcellular resolution. Optical sensing stands for detecting the nuclear size distributions or biomolecular information in tissue without the true representation of the targeted tissue’s morphology. In this review, we will in turn introduce and summarize these technologies according to this classification.

As one of the novel endoscopic optical imaging techniques, hyperspectral imaging currently enhances image contrast by altering the illumination of conventional endoscopes through wavelength dispersion devices [18]. In contrast, other techniques are implemented in the form of a miniaturized probe, which is feasible for easy deployment in more specific medical scenarios. Probe-based imaging systems have two available operation modes:

(1) Through the working channel to the distal end of the therapeutic endoscope. In this way, endoscopists can simultaneously obtain information from both the wide-field imaging system and the probe.

(2) Working alone. Especially, when endoscopists detect the narrow-body cavities such as pancreatic duct and biliary tract.

Moreover, these techniques can also be categorized based on the way how light interacts with tissues while detecting, such as reflection, absorption, elastic scattering, inelastic scattering, and fluorescence [14] (Fig. 1b). Table 1 summarizes all the endoscopic diagnostic techniques in this review.

**Table 1:** Summary of each endoscopic diagnostic technology

Technique	Excitation spectra	Frame rate (Hz)	Achievable spatial resolution (μm)		Penetration depth (mm)	Functional information		Real morphologic image	Whether the probe is in direct contact with the tissue surface while operating
HSI	Visible & Near-IR	> 30	< 250	1			✔	✔	–
FLIM	UV-Blue	20–120	< 50	< 0.1			✔	✔	Non-contact
PAE	Visible & Near-IR	2–5	10	> 1			✔	✔	Contact
OCT	Near-IR	< 50	< 3	2–4			✘	✔	Non-contact
CARS	Near-IR	0.01–0.2	< 3	> 1			✔	✔	Non-contact
TPEF/ SHG	Near-IR	1–8	< 1	0.2–0.3			✔	✔	Contact or non-contact
3PEF/ THG	Near-IR	0.05–0.1	1	0.2–0.7			✔	✔	Contact or non-contact
DRS	Visible	0.02–2	–	< 1			✘	✘	Contact
LSS	Visible	0.02–0.05	–	< 1			✘	✘	Non-contact
a/LCI	Near-IR	1–4	–	< 0.5			✘	✘	Contact
RS	Near-IR	0.1–10	–	> 1			✔	✘	Contact

HSI: hyperspectral imaging; FLIM: fluorescence lifetime imaging microscopy; PAE: photoacoustic endoscopy; OCT: optical coherent tomography; CARS: coherent anti-Stokes Raman scattering; TPEF: two-photon excited fluorescence; 3PEF: three-photon excited fluorescence; SHG: second-harmonic generations; THG: third-harmonic generations; DRS: diffuse reflectance spectroscopy; LSS: light-scattering spectroscopy; a/LCI: angle-resolved low coherence interferometry; RS: Raman spectroscopy

## Endoscopic biochemical optical imaging

### Hyperspectral imaging

Hyperspectral imaging (HSI) is a hybrid modality for optical diagnostics. It obtains spectroscopic data from an image and renders it in image form. With conventional spectroscopy, the spectral range can be recorded continuously, but for only a single analyte spot. By combining the concept of spectroscopy and digital imaging, the HSI allows the recording of the entire emission spectrum for every pixel on the entire image [19–21]. The HSI systems can provide a 3D “data tube” of spatial and spectral information of the whole image at each wavelength of interest.

On the other hand, HSI can be used for the spatial mapping of tissue morphology and physiology [22]. Likewise, it is possible to visualize the entire spatial information for a given wavelength. With spatial information, the source of each spectrum on samples can be located, which makes it possible to probe the light interactions with pathology more completely. Thus, the spectrum emitted from each pixel in the images enables HSI to identify various pathological conditions [18].

In the HSI system, visible and near-infrared (400–2500 nm) light [23, 24] is used to transmit information about molecular expression in healthy and diseased tissues. In this way, HSI generally covers a contiguous portion of the whole light spectrum with more spectral bands (up to a few hundred) and higher spectral resolution than multispectral imaging (such as RGB color cameras) [18]. Therefore, HSI may capture subtle spectral differences under different pathological conditions, while conventional multispectral imaging may miss important spectral information for diagnosis. Furthermore, a spectral signature can be constructed as a curve that links light with the target region. Then, the spectral signature is utilized as an indicator of the different biochemical components from various tissues which helps to discriminate healthy from abnormal tissue [25, 26]. In other words, HSI can identify various pathological states by extracting the spectral reflectance curve information of the pixels in the image and analyzing the tissue situation accordingly. Thus, HSI is promising as an ancillary and non-invasive method for distinguishing normal, precancerous, and cancerous cells [27].

To prove the applicability of hyperspectral imaging for the in vivo discrimination between healthy and dysplastic tissues, Arnold et al. [28] developed a hyperspectral video endoscopy system with electronically controlled acousto-optical tunable filters (AOTF) to differentiate larynx tumor through a combined classifier. Moreover, Gerstner et al. [29] implemented the HSI into endoscopy to visualize human larynx in vivo by utilizing a tunable light source for illumination. Moreover, Gu et al. [30] developed a flexible gastroscopy system capable of obtaining in vivo hyperspectral images of different types of stomach mucous diseases. Certain illumination bands, generated with a filter wheel, were assigned to color components of an enhanced image of the object with an appropriate band selection algorithm based on the dependent weight of information. Thus, images with higher color tone contrast could be attained to enhance visualization of gastric lesions. Furthermore, HSI can contribute to a more accurate determination of tumor boundaries, facilitating a complete resection of the tumor tissue [26, 31].

However, HSI is confronting notable challenges. First, the intra- and inter-patient variability of hyperspectral imaging data must be handled [24]. Second, the acquisition speed of high-resolution HSI datasets. Up to now, the HSI systems can not realize real-time detection. Third, more advanced classification algorithms are required to enable better discrimination among healthy, premalignant, and malignant tissue. Lastly, more precise delineation of cancer margins for image-guided biopsy and surgery is demanded [25].

### Fluorescence lifetime imaging

The fluorescence intensity distribution is not only proportional to the concentration of the fluorophores in the tissue but also depends on fluorescence lifetime. The fluorescence lifetime refers to the average time that the molecules remain in the excited electronic energy state before returning to its ground state via the emission of fluorescent light, which is somewhat similar to the radioactive decay of an unstable atom. Thus, fluorescent light from a sample does not instantly cease when the excitation light is extinguished. Instead, it decays away over a period of several nanoseconds as excited fluorophore molecules in the sample return to their ground state. The fluorescent molecule in the excited state emits fluorescence. It releases energy during the de-excitation to the ground state. The attenuation of the fluorescence intensity of the excited state fluorophore can be expressed as a single exponential function by a mathematical expression as follows:

1 $$I\left(t\right)={I}_{0}{e}^{-\frac{1}{\uptau }}.$$

In the Eq. (1), *I* (*t*) is the intensity measured at time t after the sample is excited by the light pulse; *I*_0_ is the intensity at *t* = 0; τ is the average fluorescence lifetime and is a characteristic value of the molecule, and is defined as the time required for the fluorescence intensity to decay to 1/e (37%) of the initial. The emission of fluorescence is a statistical process; few fluorescent molecules emit fluorescence just at the moment of τ (fluorescence lifetime). Hence, the fluorescence lifetime only reflects the time required for the fluorescence intensity to decay to its initial value of 1/e.

**Fig. 2:**
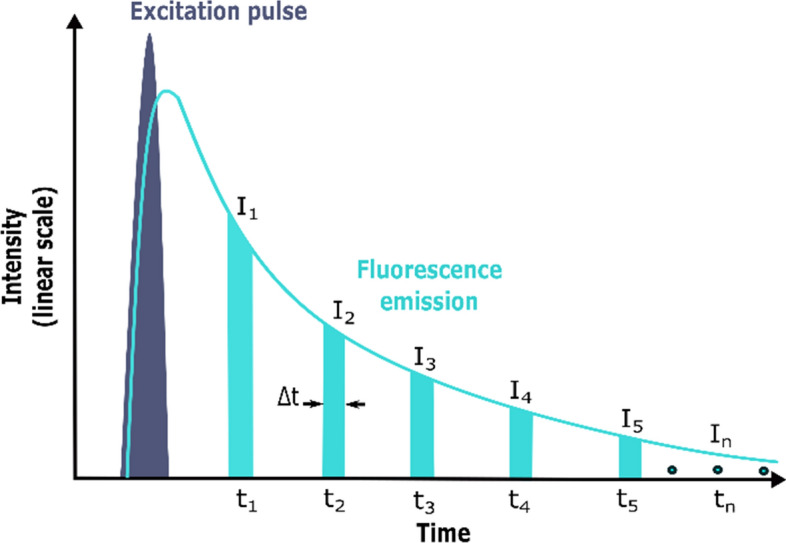
The schematic diagram of the principle of time-gated detection for FLIM. After exciting an ultrashort laser pulse, a set of fluorescence intensity images are obtained by strobing the image intensifier or CCD camera at different times (time window). When conditions permit, multi-gated detection is usually adopted, that is, multiple time windows (*t*_1_, *t*_2_, *t*_3_,…,*t*_n_) are selected to obtain multiple intensity images (usually 5 to 10). Using the formula (1), the fluorescence lifetime of each point on the sample is calculated point by point to capture the fluorescence image. The excitation light pulse is in dark blue, with the fluorescence emission in light blue. Currently, most of the devices available are capable of a minimum time gate of 3–5 ns, much faster devices, exhibiting a minimum time gate down to 80–100 ps, have been developed recently. Typical fluorescence decay times of organic compounds fall between a few hundreds of picoseconds and several nanoseconds [34]. Δt: gate width

Fluorescence lifetimes are affected by the transfer of energy from a fluorophore to its surroundings (non-radiative decay), so FLIM is capable of mapping tissue biochemical changes, including pH, O_2,_ Ca^2+^, nicotinamide adenine dinucleotide (NAD), collagen, etc. [32, 33]. Therefore, fluorescence lifetime can be invoked as a contrast mechanism for imaging, and this is achieved in fluorescence lifetime imaging microscopy (FLIM), for which the fluorescence lifetime is determined for each pixel in a field of view [34]. The fluorescence lifetime can be measured in the time and frequency domain [35]. Time-domain measurements are the most intuitive because they involve direct measurement of the decay of fluorescence intensity over time. Moreover, time-domain fluorescence lifetime measurement is mainly implemented by four conventional methods, including time-correlated single-photon counting (TSCPC) [36–38] time-gated detection (Fig. 2) [39, 40], streak-FLIM [41] and pulse sampling techniques [42]. By contrast, frequency-domain FLIM uses modulation technique, which measures the phase shift and demodulation during excitation with sinusoidally modulated light to perform FLIM [43].

In the last few years, several endoscopic systems based on FLIM designs have been proposed [38, 39, 44–46]. Thereinto, McGinty et al. [47] developed a compact, clinically deployable instrumentation which achieved wide-field fluorescence lifetime images of unprecedented clarity to intrinsic tissue autofluorescence. Statistically, significant contrast was observed between cancerous and healthy colon tissue with excitation at 355 nm. Additionally, FLIM image was acquired ex vivo at near video rate, which was an essential step towards real-time FLIM for screening and image-guided biopsy applications. In 2013, Sun et al. [48] proposed an in vivo clinically compatible FLIM system, which consisted of a rigid fiber-bundle endoscope proving a field of view of 4 mm. Intraoperative imaging of head and neck squamous cell carcinoma was performed in 10 patients at 337-nm excitation and fluorescence collected in the 435–485 nm range to facilitate the margin demarcation of the tumor. Shorter average lifetime (1210 ± 40 ps) was exhibited from head and neck squamous cell carcinoma than the surrounding healthy tissue (1490 ± 60 ps).

More recently, Ning et al. [49] proposed a wide-range pH-sensitive Yb^3+^ porphyrinate-based molecular probe, which utilized time-resolved FLIM in the near-infrared region of 900–1700 nm. The Yb^3+^ probe showed increasing NIR emission and lifetime with pKa values of ca. 6.6 from pH 9.0 and 5.0. Also, the system displayed an elongated lifetime from about 135 to 170 ms at lower pH values (5.0–1.0) due to aggregation and reduced exposure to water at low pH values. What is more, the probe could monitor a wide range of in vivo gastrointestinal pH values in mice models, which showed that lifetime contrast might be necessary for preclinical imaging. Furthermore, due to the long lifetime (millisecond scale) of lanthanide, the Yb^3+^ probe could quantitatively, dynamically, and in situ monitor real-time pH changes in gastric cells, with deep penetration, good reversibility, and high spatiotemporal resolution.

Further studies will be necessary to optimize and refine the FLIM technique to maximize the contrast available for a given tissue or tumor. And more promising early findings should be replicated in vivo to early approval of clinical applications [47].

## Endomicroscopic imaging techniques

Unlike wide-field imaging techniques that detect morphologic changes visible to the unaided eye, novel endomicroscopy facilitates the visualization of cellular-scale or subcellular-scale objects in tissue, such as goblet cell, epithelium cells, organelles (nucleus and cytoplasm), etc. Compared with commercial endomicroscopy, these novel emerging techniques can provide high-definition three-dimensional images with better penetration. Thus, they have the potential to challenge traditional ex vivo histology by executing accurate in vivo “optical biopsy”. Table 2 summarizes the differences between wide-field technologies and endomicroscopy.

**Table 2:** The comparison of properties between the endoscopic wide-field imaging and endomicroscopy

	Wide-field imaging	Endomicroscopy
Resolution	Around 100 μm	0.5–20 μm
Field of view	80–140°	Ø0.3–Ø3 mm
Detection target	Macroscopical objects in tissue	Tissue structures in cellular and subcellular scale
Corresponding techniques	Traditional white-light imaging Narrow-band imaging Linked color imaging i-scan	Endocyto Confocal laser endomicroscopy Photoacoustic endoscopy Optical coherent tomography Coherent anti-Stokes Raman scattering Multi-photon endomicroscopy

### Photoacoustic endoscopy

Currently, endoscopic ultrasound (EUS) [50] is the only clinically available tomographic endoscopic tool used to diagnose various diseases [51, 52]. EUS cannot receive high-resolution vasculature information of the suspicious region, owing to the limited detectable depth and the operating mechanism.

**Fig. 3:**
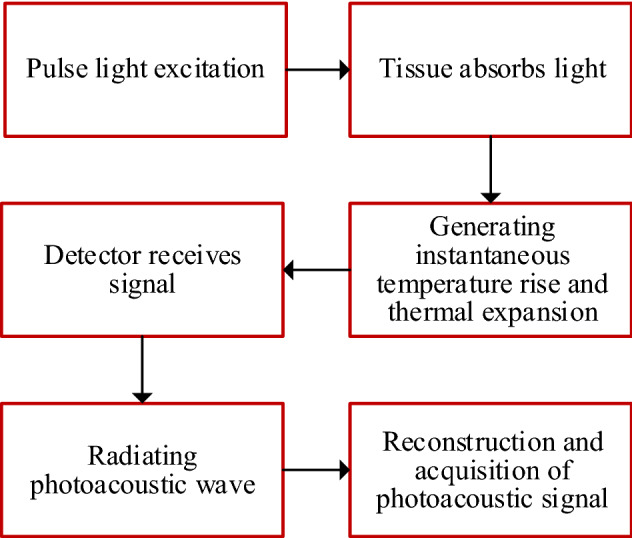
Photoacoustic effect and the imaging process

Although photoacoustic spectroscopy and simple imaging were developed in the 1970s, photoacoustic imaging only recently became famous in biomedical research [53]. The advent of photoacoustic endoscopy (PAE) aims to overcome the EUS’s limitations by adapting the photoacoustic effect. Photoacoustics can be described as a laser-induced ultrasound [54]. Short light pulses (nanosecond range) are absorbed by tissue absorbers accordingly (such as endogenous contrast: hemoglobin, melanin and exogenous contrast: methylene blue [55]). Then a transient temperature increase is generated, resulting in local thermoelastic expansion effect, which gives rise to ultrasonic waves [56]. After that, the ultrasonic wave signal is recorded by nearby detectors [57]. Figure 3 illustrates the imaging process of PAE. Photoacoustic imaging has the primary advantages of beneficial penetration (from millimeters to centimeters) and high spatial resolution because it overcomes the high degree scattering of optical photons in biological tissue.

Compared with NBI, CLE, and OCT [58], PAE provides functional optical contrast with high spatial resolution while maintaining the benefits of greater penetration depth of traditional ultrasound endoscopy [59]. Particularly, PAE embodies photoacoustic tomography (PAT), which is cross-sectional or three-dimensional imaging using the photoacoustic effect. Thus, PAE can offer in vivo 3D volumetric images of biological tissues with high spatial resolution and stark tissue-optical contrast [60]. Thus, characteristics of the targeted area, like architectural vascular features and dynamic changes of tumors, can be clearly observed from the 3D images [61].

The prototype PAE was based on a scanning mirror system that deflected both the light and the ultrasound. The mirror was integrated into a mini-probe that could be inserted into the working channel of the endoscope. Viator et al. [54] first developed a photoacoustic endoscopic probe for 1D sensing. Later, Lim et al. [62] conducted an ex vivo clinical trial using photoacoustic imaging (PAI) in patients undergoing endoscopic mucosal resection (EMR). The esophageal microvascular pattern could be characterized by creating a 3D reconstruction of the full ex vivo tissue volume. Moreover, many in vivo preclinical trials on animal tissue have been finished recently [63–67], which demonstrated the integrated in vivo imaging capability of the PAE-EUS system. He et al. [68] proposed a hybrid optical-resolution (OR) and acoustic-resolution (AR) photoacoustic endoscope, which was appropriate for improving the endoscopic depth and resolution range. In this system, the optical resolution achieved the order of 13 μm, which is feasible to distinguish the numerous smaller vessels ex vivo. However, the smaller vessels were not visible on the AR mode, with a reported lateral resolution 250 μm. In addition, Yang et al. [67] initially created an optical-resolution photoacoustic endomicroscopy (OR-PAEM) system. The transverse resolution and radial resolutions were 10 µm and 50 µm, respectively, with an imaging frame rate up to 2 Hz. The typical imaging depth of their system was 1 mm. However, the diameter of the probe was 3.4 mm, larger than the work proposed by Bai et al. [69] whose probe-diameter was 1.1 mm with transverse resolution as fine as 19.6 μm. Furthermore, Lin et al. [70] used a flexible coil to transmit the rotational torque from the rotary stage, which enabled a more extensive 360° field-of-view imaging at a 5-Hz B-scan rate in vivo.

To conclude, PAE has great potential for in vivo endoscopic applications, such as precancer detection, accurate diagnosis of submucosal abnormalities, and in situ characterization of diseased tissues. Endogenous or exogenous contrast agents may improve the ability of endoscopic imaging, resulting in the earlier and more accurate detection of malignant and premalignant lesions. The wealth of experience gained in preclinical studies as well as recent technological progress provides a solid foundation for photoacoustics to advance into mainstream use [55].

Nonetheless, further refinement of photoacoustic imaging is necessary for broader dissemination, including stable laser systems, user-friendly imaging devices, algorithms and compatible software that can robustly analyze imaging data to extract diagnostically relevant quantity. Moreover, the most apparent limitation remains the penetration depth of light. The near-infrared light is not sufficient to visualize targets beyond a few centimeters under the tissue surface [55]. Additionally, the imaging speed of PAE systems is relatively low, so there is still a long way to implement real-time detection.

### Optical coherent tomography

Initially proposed in the early 1990s [71], optical coherent tomography (OCT) is a promising new tomography technology. OCT is another considerable breakthrough for medical imaging after computerized tomography (CT) and nuclear magnetic resonance (MRI). Similar in principle to ultrasonography [72], the OCT system based on the technique of low coherence interferometry. A light source emits low coherence broadband signals, while a beam splitter splits the light into two identical beams, one directed to the tissue and the other to a mirror. Then, the system utilizes an interferometer to combine the back reflection or scattering signal coming back from the tissue and the signal from the mirror [71]. The interference between the two beams is measured, then an image is created by analyzing single points in different depth layers within one axis [73].

One of the OCT’s most significant advantage is the axial resolution (δ_z_), which mainly depends on the coherence length of the light source (*l*_c_). Higher axial resolution can be obtained when using a common objective lens with a small numerical aperture. For a Gaussian light source, it can be expressed as follows:

2 $${\updelta }_{z}=\frac{{l}_{c}}{2}=0.44\frac{{\lambda }_{0}^{2}}{\Delta \lambda }$$

According to formula (2), under the condition that the spectral bandwidth (λ_0_) of the light source is constant, there is a simple inverse relationship between the axial resolution and the bandwidth of the light source (Δ*λ*). But in practice, the axial resolution of OCT is ultimately limited by the available broadband light sources, detectors, and passive optical devices (such as couplers, circulators) and dispersion effects in optical systems. However, with the continuous progress of technology, it can be found that the axial resolution of OCT is also continuously improved.

Moreover, as long as the scattering effect decreases with increasing wavelength, longer wavelengths in the near-infrared region (from 800 to 2500 nm) can be utilized in OCT to realize high-resolution images with deeper penetration (2–4 mm) instead of the visible-range wavelength that everyday optical microscopy use. Similar to traditional optical microscopes, the lateral resolution of the OCT depends on the focus state of the detection beam, which is characterized by the numerical aperture (*f*) of the objective lens:

3 $${\updelta }_{x}=\frac{{\lambda }_{0}}{\pi }=\frac{f}{D}$$

where D is the diameter of the light spot at the focusing point of the sample arm. Higher numerical aperture microscope objectives can be used to improve the lateral resolution of the OCT system.

By rotationally scanning, the label-free two-dimensional (2D) or three-dimensional (3D) structure image of the biological tissue can be obtained. Thus, the non-contact, non-invasive tomography of living tissue is realized [74]. Any physical property that alters the amplitude, phase and polarization properties of the sample light can be used to extract diagnostic information.

Acted with low-numerical-aperture lenses, the mechanical components of OCT can be miniaturized. Furthermore, it is easy to combine with endoscopes, thanks to the no-touch and fiber-based feature of broadband low coherence light source and Michelson interferometers [75]. Therefore, the miniaturizing design of the scanning probe determines the application and development of the OCT-based endoscopy. Combining the advantages of both OCT and conventional endoscopy, OCT-based endoscopy eliminates the shortcomings of OCT: (1) cannot detect human internal organs in vivo, and (2) cannot diagnose small lesions as well as early carcinomatous change [76].

The first endoscopic OCT scanning probe was proposed by Tearney et al. [77] In the subsequent works, the scan speed of the probes was improved (up to 50 frames per second by Wang et al. [78]), and the diameter of the probe decreased significantly (even up to 160 μm by Lee et al. [79]). In addition, the lateral and axial resolution of endoscopic OCT image is continuously improving [80]. Pahlevaninezhad et al. [81] recently developed an endoscopic OCT catheter that connecting to a Fourier-domain OCT system [82]. By integrating a metalens which can modify the phase of incident light at sub-wavelength level, near-diffraction-limited imaging (with a resolution of 6.37 µm in tangential and 6.53 µm in sagittal) was achieved through negating non-chromatic aberrations. Additionally, the trade-off between transverse resolution and depth of focus was eased (effective depth of focus was achieved as 211 µm in tangential and 315 µm in sagittal).

**Fig. 4:**
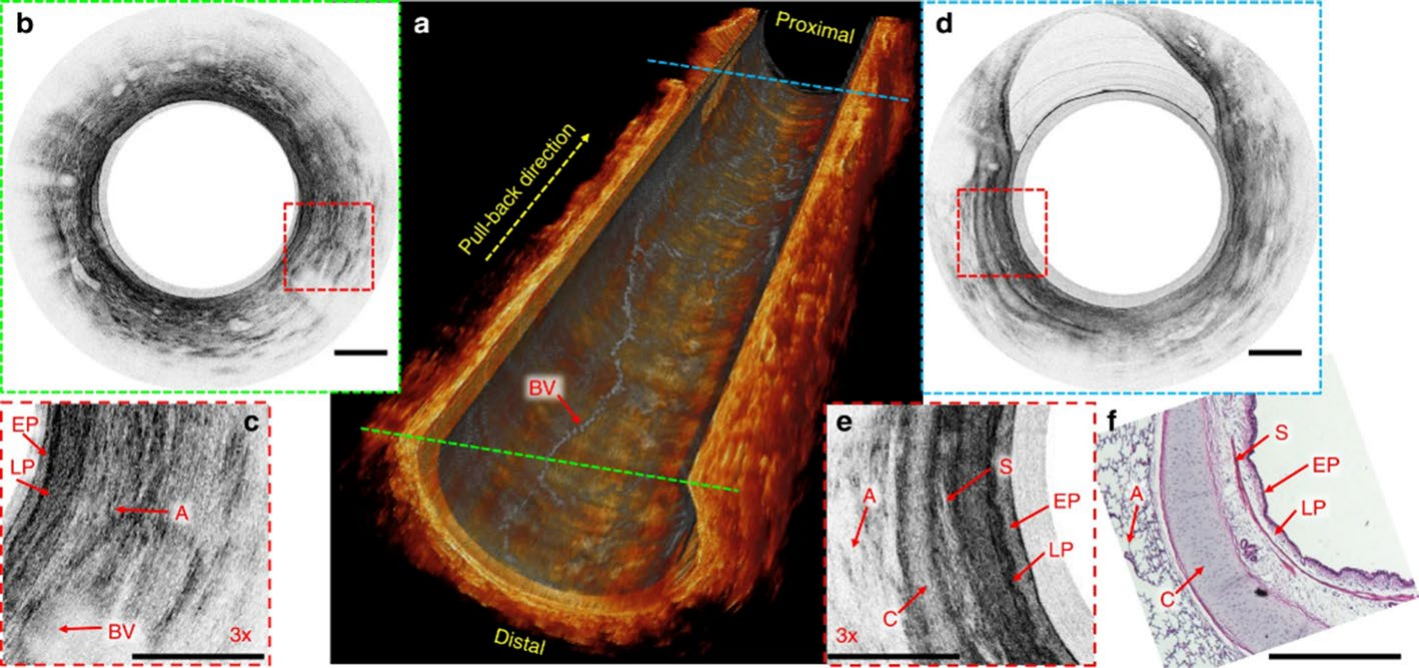
In vivo endobronchial imaging of sheep small airways. **a** Cut-away view of a reconstructed three-dimensional (3D) image of an 8 mm-long sheep small airway with the en face projection view of blood vessels overlaid on the inner surface of the three-dimensional (3D) image. **b** Representative in vivo 2D image of the peripheral sheep airway corresponding to the cross section indicated with the green dashed line in **a**. **c** 3× enlarged view of the area boxed with red dashed lines in **b**. **d** Representative in vivo 2D image of the sheep small airway, which is relatively more towards the proximal side (indicated with the blue dashed line in **a**). **e** 3× enlarged view of the area boxed with red dashed line in **d**. **f** Corresponding hematoxylin and eosin (H&E) histology. A: alveoli; BV: blood vessel; C: cartilage; EP: epithelium; LP: lamina propria; S: smooth muscle. All scale bars are 250 µm. Adapted with permission from Ref. [85]. Copyright 2017 Springer Nature Publishing

Furthermore, by effectively reducing the operating central wavelength in the OCT system to 800 nm, compared with the previous work at 1300 nm [83, 84], Yuan et al. [85] developed an ultracompact (520 µm in outer diameter and 5 mm in rigid length) and super-achromatic endoscopic OCT microprobe made with a built-in monolithic fiber-optic ball lens. Their system could image sheep small airways.in vivo with 1.7-µm axial resolution and 6-µm transverse resolution in real-time (about 5 fps) (Fig. 4). Later on, Mavadia-Shukla et al. [86] presented a distal scanning OCT probe delivered through a conventional endoscope’s working channel to perform in vivo imaging at 50 frames per second while maintaining an ultra-high axial resolution (2.4 µm) with higher contrast.

At present, the endoscopic OCT system has only been adopted into clinical practice in the cardiovascular department. The existing clinically available OPTIS System (Abbott, USA) offers physicians an efficient way to optimize percutaneous coronary intervention (PCI) and to image coronary arteries. However, instead of characterizing lesions, this OCT system mainly focuses on helping physicians improve stent size and placement decisions according to the measured shape of coronary artery due to its relatively low imaging resolution. Ding et al. [87] proposed a lensless OCT probe that also had the potential to be used in cardiovascular investigations. The probe is only 340 μm in diameter and 6.37 mm in length. It was composed of a segment of large-core multimode fiber, a segment of tapered multimode fiber and a length of single-mode fiber. A controllable output beam can be designed by a simple adjustment of its probe structure parameters (PSPs), instead of the selection of fibers with different optical parameters. An effective imaging range of ∼0.6 mm with a full width at half-maximum beam diameter of fewer than 30 μm was realized with high sensitivity by the probe.

Although OCT helped identify cellular structures of tissue, it was incapable of offering functional information on the region of interest and distinguishing different cytological lesions [88]. Additionally, it was challenging to affirm the correlation between morphological changes visualized in the OCT images and their corresponding histology [89], which could render endoscopic OCT ineffective in the provision of diagnosis [90]. Thus, further studies on the improvements in the resolution, frame rate, and depth of detection of endoscopic OCT imaging systems are warranted [80, 91–96].

### Coherent anti-Stokes Raman scattering

Coherent anti-Stokes Raman scattering (CARS) microscopy has been demonstrated as a promising technology for label-free biomedical imaging [97–101]. The CARS microscopy offers many advantages, including (1) chemical contrast based on Raman vibrational activity, (2) high sensitivity and rapid acquisition rates due to the coherent nature of the CARS process, (3) and sub-wavelength spatial resolution [102]. Compared to Raman spectroscopy, CARS employs multiple photons to probe the molecular vibrations and produces a signal of coherent emitted waves. As a result, the CARS signal is orders of magnitude stronger than spontaneous Raman emission and can provide much faster imaging.

**Fig. 5:**
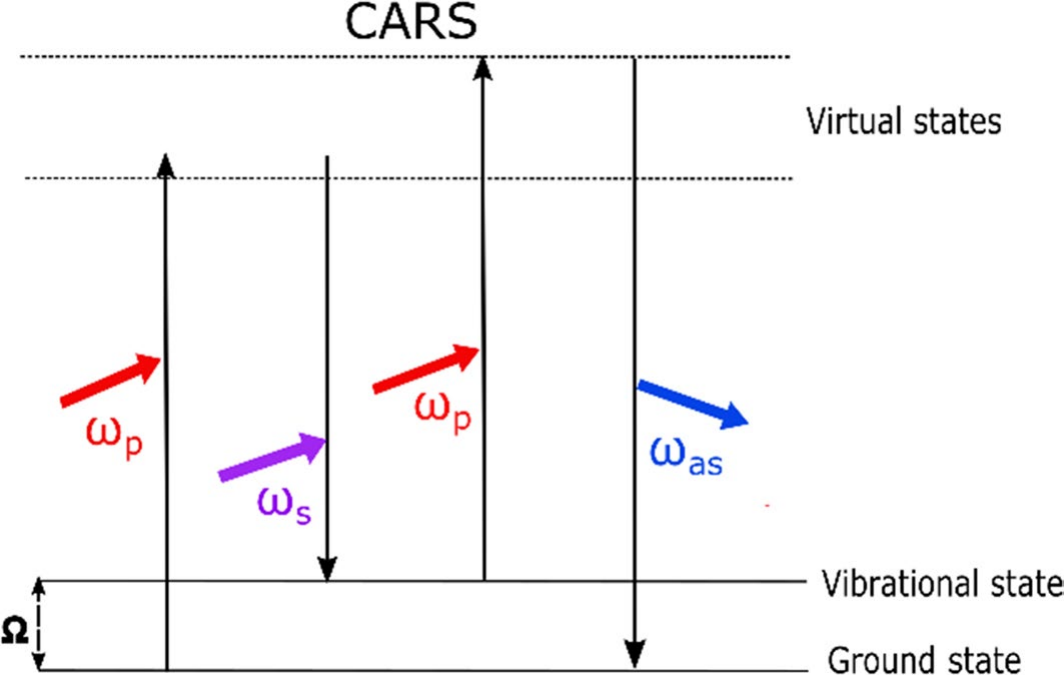
Schematic diagram of CARS energy

CARS requires two laser frequencies called “pump” (*ω*_p_) and “Stokes” (*ω*_s_). When the difference of pump and Stokes’s frequency, Δ*ω* = *ω*_p_-*ω*_s_, is set to that of vibrational resonance, Ω, a strong optical response occurs at the “anti-Stokes” frequency: *ω*_as_ = 2*ω*_p_-*ω*_s_ (Fig. 5). The CARS signal is derived from the third-order nonlinear susceptibility, *χ* (3), of a material, which is significantly enhanced when the different frequency is tuned into a resonance. In addition, CARS requires a high peak-power for efficient signal generation, and tunable picosecond laser sources are optimal for CARS imaging applications as they achieve high spectral resolution and optimal signal to background ratios [102].

Liu et al. [103] developed a CARS prototype endomicroscopy using a fiber bundle with the polarization four-wave mixing (FWM)-suppressing scheme. The highly improved collection efficiency and isolation of excitation laser dramatically enhance the image quality. Also, with a broadband dual-wavelength waveplate (DWW), spectroscopic CARS imaging capability could be achieved in the FWM-suppressing scheme. 3D scanning capability was also demonstrated. Recently, Hirose et al. [101] created a rigid endoscope toward nerve-sparing robot-assisted surgery which achieved 0.91% image distortion and 8.6% non-uniformity of CARS intensity in the whole field of view (650 µm diameter field of view (450 µm × 450 µm square image)). An image with a spatial resolution of 2.91 µm was obtained by scanning the beams with galvanometer mirrors and detecting the backscattered CARS signal with a photomultiplier tube. Furthermore, the imaging time was reduced to several seconds by increasing the CARS intensity. However, although the above prototypes capable of collecting CARS signals have been developed, future expansion in delivering in vivo CARS imaging in various medical specialties are still awaited.

### Multi-photon endomicroscopy

**Fig. 6:**
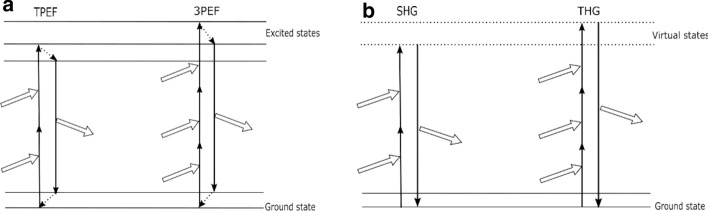
Schematic representation of MPE. **a** TPEF and 3PEF. **b** SHG and THG Hollow arrows represent incoming and radiated photons, and dashed arrows represent non-radiative relaxation processes

Multi-photon imaging is a promising technique for label-free imaging of biological samples [104]. The multi-photon effect refers to the phenomenon that a molecule simultaneously absorbs or scatters two or more nonlinear photons with the same frequency, relaxes to the ground state after reaching the high energy state, and emits shorter wavelengths which can be used for biomedical imaging. Compared to single-photon imaging, multi-photon imaging uses a near-IR light source that is able to create sufficient peak energy to produce multi-photon effect while keeping average energy low enough to minimize specimen damage [105]. There are several nonlinear imaging modalities, such as two-photon excited fluorescence (TPEF), three-photon excited fluorescence (3PEF), second-harmonic generation (SHG) and third-harmonic generation (THG) imaging (Fig. 6). They can provide valuable information about the biological samples’ intrinsic properties, which can be employed in diagnosis applications [106, 107].

It is challenging to acquire high-resolution images from tissue in the deep layer, for the high degree scattering caused by discontinuous refractive index and heterogeneous constituents. Despite that, the near-IR owns deeper penetration depth and causes less damaging than shorter wavelengths [108]. Thus, the infrared light can be selected as the excitation light source for multi-photon imaging, which effectively reduces the scattering of excitation light and gets a high signal-to-noise ratio by avoiding the interference of single-photon autofluorescence.

Furthermore, no pinhole is required to prevent out-of-focus fluorescence from reaching the detector in MPE, because excitation occurs only at the excitation spot, which results in the optical sectioning inherent to nonlinear imaging techniques. MPE owns the advantages of strong penetrability, high resolution, low phototoxicity, and no need to stain the tissue [109]. MPE can ex vivo or in vivo image and detect early invasion of cancer in reconstructed 3D photos as well as 2D views [110]. The critical challenge of realizing MPE is how to miniaturize the system into a compact and lightweight distal probe while keeping the general performance. The following reviews research works on various types of MPE.

#### TPEF

The desktop two-photon microscopy was pioneered by Denk and Strickler in 1990 [111]. Subsequently, endoscopic applications of TPEF have been explored. Huland et al. [112] used a composite GRIN lens system with a length of 8 cm and a fast-moving robotic arm to create a portable rigid endoscopic system for multiphoton in vivo imaging of deep tissue. They used it for imaging the liver and kidney of live mice. Moreover, the inner surface of the colon was imaged.

Brown et al. [113] demonstrated the first work that used a compact and flexible TPEF endomicroscope to acquire in vivo fluorescence images of unstained tissue from a live subject (liver, kidney, and colon from an anesthetized rat). The device delivered femtosecond pulsed 800-nm light from the core of a raster-scanned dual-clad fiber (DCF). The light was focused by a miniaturized gradient-index lens and projected onto the tissue. In this work, images under field-of-view of 115 μm by 115 μm with 4.1-Hz frame rate were obtained. In addition, lateral and axial two-photon resolutions measured 0.8 and 10 μm (full width at half maximum), respectively, in the image plane.

Later, a 2.2-mm-outer-diameter probe was developed by Ducourthial et al. [114], which also allowed simultaneous TPEF and SHG imaging at 8 frames per second. They successfully got images with penetration depth greater than 300 μm below the surface of an anesthetized mouse kidney. And the field (FOV) of the system was up to 450 μm, while transverse and axial resolutions reach 0.8 μm and 12 μm, respectively.

**Fig. 7:**
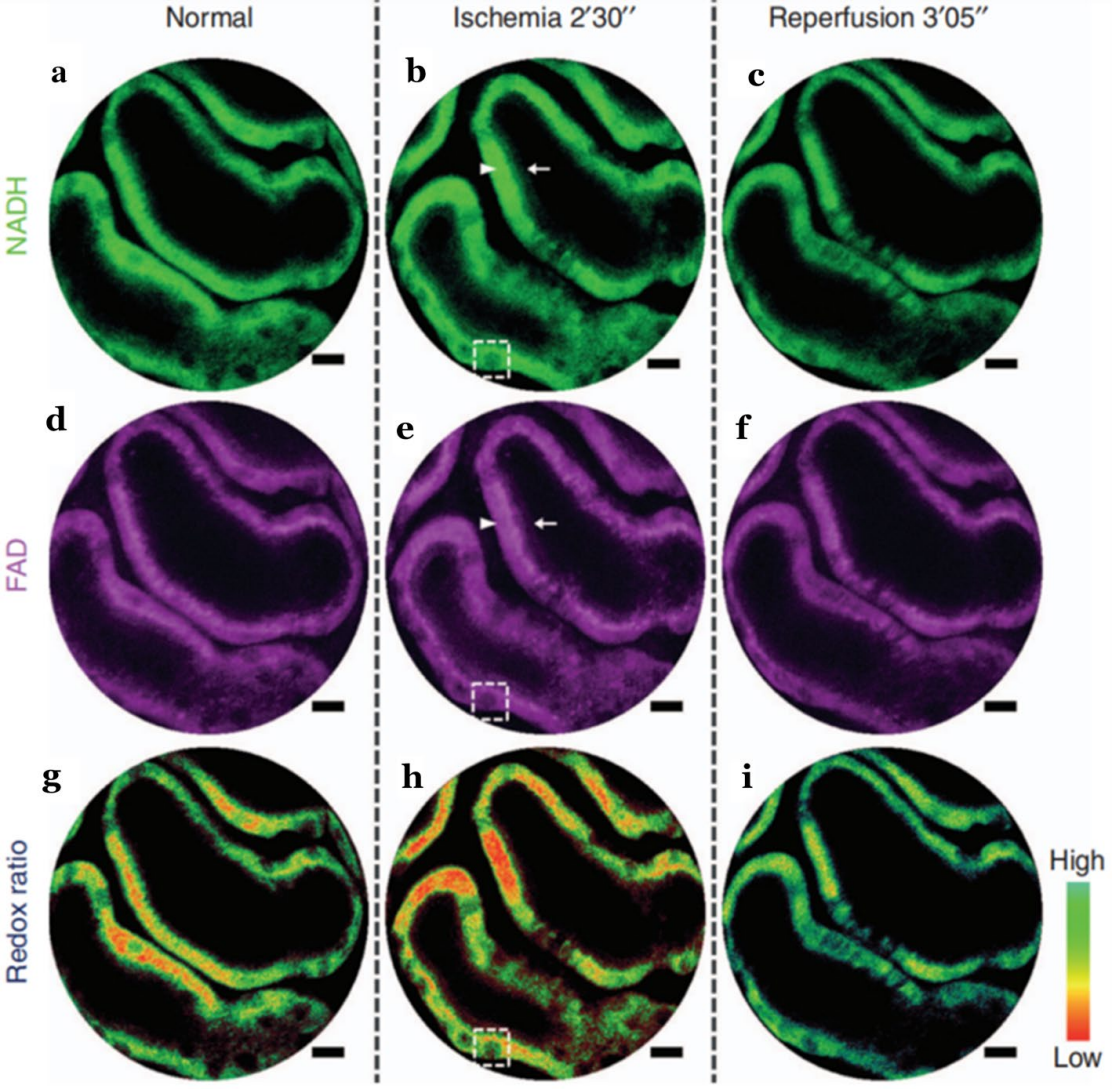
Endomicroscopy 2PF redox imaging of a mouse kidney ischemia–reperfusion model in vivo. Top row **a**–**c** 2PF intensity images from the NADH detection channel (417–477 nm); middle row **d**–**f** 2PF intensity images from the FAD detection channel (496–665 nm); bottom row **g**–**i** 2PF intensity images color-coded by the measured optical redox ratio defined as FAD/(FAD + NADH), where more reddish (greenish) color corresponds to an increased (reduced) concentration of NADH and thus a reduced (increased) redox ratio. The dark round-to-elliptical spots scattered along the renal tubule wall (dashed squares) correspond to the nuclei of renal tubular cells. Each column corresponds to one specific time point: normal (left), 2 min 30 s post-ischemia (center), and 3 min 05 s post-reperfusion (right). 2PF, two-photon fluorescence; Scale bar = 10 μm. Adapted with permission from Ref. [115]. Copyright 2017 Springer Nature Publishing

In 2017, Liang et al. [115] pushed out the first fiber-optic endomicroscopy platform, which achieved subcellular resolution. The platform was implemented to perform label-free two-photon metabolic imaging of living tissue in vivo. With a customized dispersion compensating fiber, the image quality of the system can compare favorably with a standard bench-top two-photon microscope (The spatial resolution of the endomicroscopy was 0.7 μm laterally and 6.5 μm axially, and a frame can be acquired within only 0.38 s). In their experiment, a model of acute mouse kidney ischemia–reperfusion was performed in vivo: the left renal artery was occluded in the anesthetized mouse, the blood was induced by the venous vein (4 min), and then reperfusion was performed. Changes in the redox rate of renal cortical tubules were monitored in histological resolution by the TPEF endomicroscopy platform. And the endogenous fluorescent substances: NADH, FAD were selected. In addition, the redox ratio was expressed as FAD/(FAD + NADH). At the time of ischemia, the concentration of NADH increased, while the redox ratio decreased. Thus, endoscopic metabolic imaging was successfully achieved in the normal mouse kidney model (Fig. 7). This device has made unprecedented detection sensitivity and was capable of capturing unlabeled subcellular living structures and dynamic functional metabolic information.

**Fig. 8:**
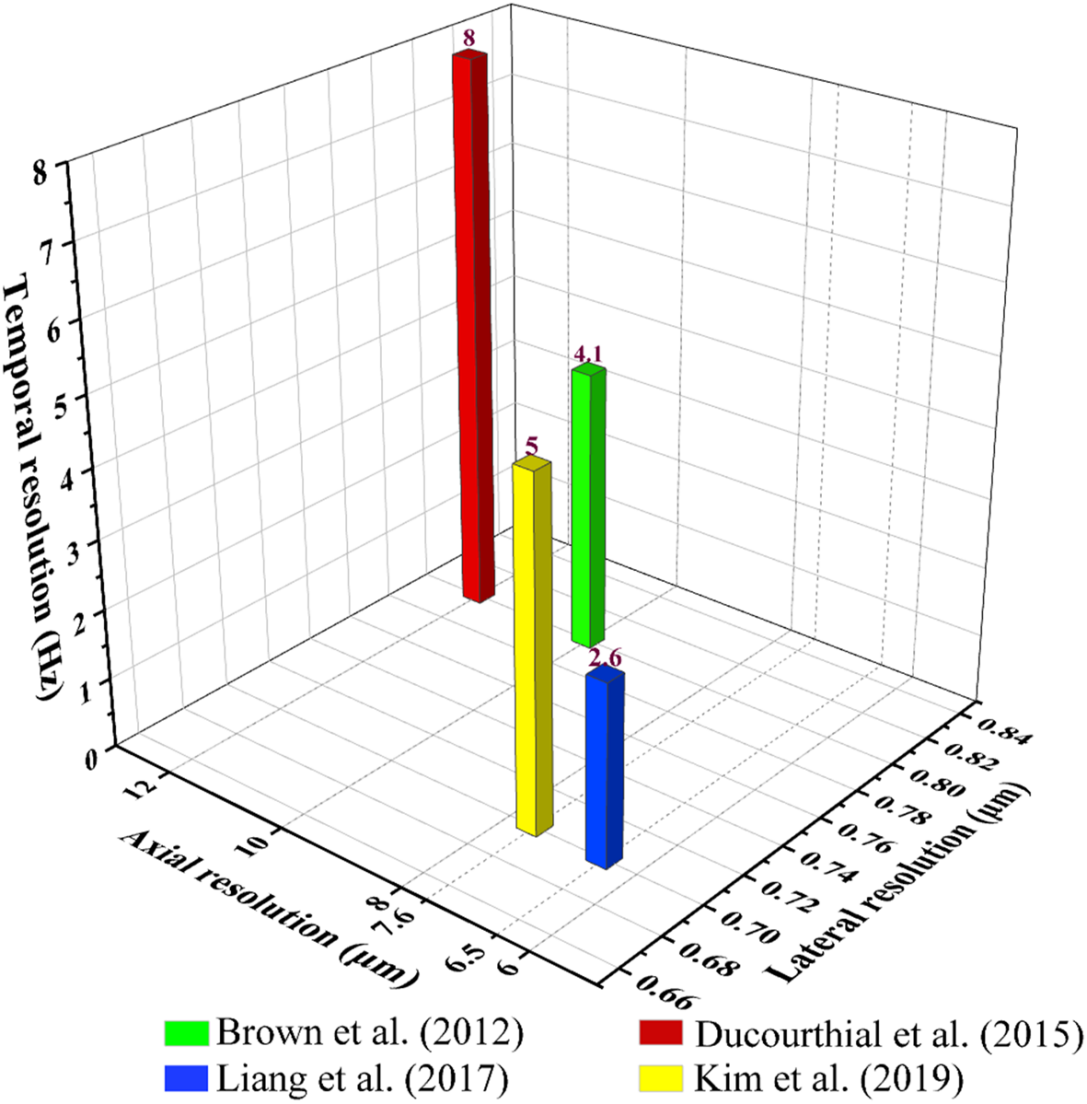
Comparison of the spatiotemporal resolution of TPEF endomicroscopes mentioned above. Its corresponding imaging spatial resolution limits the imaging speed of each system. Green, red, yellow, and blue bars in the chart represent works in Refs. [103, 104, 105, 106], respectively

More recently, a 2.6-mm-diameter TPEF probe with a high numerical aperture gradient index (GRIN) lens was developed by Kim et al. [116]. A Lissajous fiber scanner which consisted of a piezoelectric (PZT) tube and a micro-tethered-silicon-oscillator (MTSO) was installed for the separation of biaxial resonant scanning frequencies. The lateral and axial resolutions of the endomicroscopy could achieve a higher level: 0.70 µm and 7.6 μm, respectively. TPEF images of a stained kidney section and miscellaneous ex vivo and in vivo organs from wild type and green fluorescent protein transgenic (GFP-TG) mice were successfully obtained at a frame rate of 5 Hz using their system. Figure 8 compares the spatiotemporal resolution of several TPEF endomicroscopes mentioned above.

#### 3PEF

Comparing to TPEF, three photons are absorbed almost simultaneously during the excitation process in the 3PEF endomicroscopy. By utilizing an excitation light source with a longer wavelength, the scattering of biological tissues can be further overcome with reduced water absorption [117]. Thus, imaging depth could be increased, which is beneficial to 3D structural imaging.

Huland et al. [118] presented an endoscope system with a compact and portable three-photon gradient index (GRIN) lens working at 1040 nm, which was suitable for imaging tissues without staining. Its lateral and axial resolution in water were 1.0 μm and 9.5 μm, respectively. The FOV was 200 μm in diameter, and the frame rate was 2 Hz for visualizing ex vivo unstained mouse lung.

In addition, Akhoundi et al. [119] developed a compact fiber-based 3PEF which was capable of performing THG, SHG, and 3PEF imaging over a large field of view (900 µm) with a high lateral resolution (2.2 µm) and a frame rate of 15 s/frame. The image depth was increased to up to 200 µm. Furthermore, the author believed that by optimizing the laser pulse delivery, the penetration depth could be increased to 700 µm in the future prototype.

In future work, the increase of chromatic aberrations in the 3PEF optics due to the more considerable difference between the excitation and signal wavelengths should be further considered. And the fiber delivery of the energetic femtosecond pulses for three-photon excitation is needed to be concerned [118]. In addition, the imaging speed of 3PEF is too low to detect the targeted region in real time. Thus, the time-efficiency for 3D imaging is limited.

#### SHG and THG

As one of the most widely used contrast-enhancing mechanisms in optical microscopy, SHG and THG are nonlinear optical processes in which two (SHG) or three (THG) photons with the same frequency interact with a nonlinear material under investigation, are “combined”, and generate a new photon with twice (SHG) or three times (THG) the energy of the initial photons. Thus, SHG is a second-order process while THG is a third-order process.

The most significant difference between SHG/THG and TPEF/3PEF is the way the light interacts with the targeted region: In SHG/THG the photons are scattered to produce a new photon with two (SHG) or three (THG) times the energy of the incident photons. Compared with TPEF/3PEF, two photons are absorbed to produce a single fluorescent photon. Thus, heating and damage of tissue are less likely to occur due to the absence of absorption. For these nonlinear optical effects are proportional to the second or third power of the fundamental light intensity, only the light at the focal plane of the optic efficiently drives the nonlinearity [107]. The second-order nonlinear susceptibility of a medium characterizes its tendency to cause SHG. Thus, SHG can reveal unique molecular features of microscopic objects that are otherwise obscured by the ensemble-averaged measurements, especially in noncentrosymmetric structures (such as collagen), with reported tissue penetration depths between 100 and 300 μm at laser excitation in the near-infrared range.

Zhuo et al. [120] analyzed the SHG images of 72 fresh colonic biopsy specimens ex vivo and were able to quantify significant changes in the circle length of the crypt basement membranes of normal, precancerous and cancerous colonic tissues. Furthermore, Zhang et al. [121] proposed a compact fiber-optic SHG scanning endomicroscopy and its application to visualizing cervical remodeling during pregnancy. Significant morphological changes in cervical collagen were evident over the course of pregnancy. Furthermore, the compact fiber-optic SHG endomicroscopy had a resolution and image quality comparable to a bench-top microscope (0.76 × 4.36 μm (lateral × axial)).

Unlike SHG, the THG detects interfaces and inhomogeneities due to its nonlinear nature [122], and THG is generated only near the focal point. Therefore, high lateral resolution can be obtained, allowing THG microscopy to perform sectioning and to construct three-dimensional images of transparent samples.

Since all materials have non-vanishing third-order susceptibilities, THG microscopy can be utilized as a general-purpose microscopy technique [123]. Mehravar et al. [110] developed a compact label-free MPE system working at 1560 nm, which generated endoscopic images of cell nuclei and collagen. The system could acquire the SHG and THG images simultaneously. The backscattered THG, back-reflected forward THG and SHG signals were combined for detecting the tissue of Barrett's esophagus. Furthermore, the system shows the thickening of sub-epithelial (basement membrane) collagen with advancing stages of dysplasia. Additionally, the first flexible endoscopic THG imaging system was proposed by Akhoundi et al. [119]. In their work, the lateral imaging resolution was 2.2 µm, while the axial resolution was 12.7 µm.

However, the application of THG was not mature because of the instability of specialized high-energy excitation light.

## Optical sensing technologies

### Diffuse reflectance spectroscopy

Spectroscopy is not easily affected by artifacts or sampling errors, so it can provide quantitative data that are free of subjective interpretation. The light delivered to the tissue surface undergoes multiple elastic scattering and absorption, and part of it returns as diffuse reflectance carrying quantitative information about tissue structure and composition [124]. Moreover, primary scattering centers of tissue are the extracellular matrix consisting of a collagen fiber network and intracellular structures with sizes smaller than optical wavelengths [125]. And larger intracellular structures, such as the nuclei, also scatter light, with their relative contribution on increasing the backscatter direction [126].

Diffuse reflectance spectroscopy (DRS), also known as elastic scattering spectroscopy (ESS), analyzing mainly multiply scattered light, is sensitive to the bulk tissue scattering properties (the size and packing of dense subcellular components such as the nucleus, nucleolus, and mitochondria) as well as the absorption properties of hemoglobin [127, 128]. In the DRS system, a light source with broadband, typically the white light, is utilized to illuminate the sample. The reflected signal is collected and transmitted by optical fibers to the analyzing spectrometer [129]. Delivery and collection fibers are usually separated by a set distance, which allows the spectrometer to detect diffuse reflections preferentially [130]. Enlarged dense, crowded epithelial nuclei and an increased nucleus-to-cytoplasm ratio can be the primary pathological indicators of cancer, dysplasia, and cell regeneration [131, 132].

In the late 1990s, Mourant et al. [133] proposed the first DRS system to conduct clinical trials towards the gastrointestinal tract in vivo. Reflectance spectra were taken in vivo from the colon in 15 patients and the stomach in 17 patients. The slope of the spectrum in the 435 to 440 nm range was used to reasonably separate active colitis from quiescent colitis and normal colonic mucosa. When multiple linear regression was used for spectral classification, adenomatous polyps were distinguished from hyperplastic polyps with a sensitivity of 89% and a specificity of 75% [134]. Additionally, Zonios et al. [124] analyzed the data using an analytical light diffusion model, which was tested and validated on a physical tissue model composed of polystyrene beads and hemoglobin. Four parameters were obtained to tell healthy tissue from adenomatous tissue: hemoglobin concentration, hemoglobin oxygen saturation, effective scatterer density, and adequate scatterer size, which suggest that diffuse reflectance can be used to obtain tissue information about tissue structure and composition in vivo.

In addition, Lovat et al. [130] collected elastic scattering spectroscopy measurements in vivo, which were matched with histological specimens taken from identical sites within Barrett’s esophagus. A total of 181 matched biopsy sites from 81 patients, where histopathological consensus was reached and analyzed. The results were that there was a good pathologist agreement in differentiating high-grade dysplasia and cancer from other pathology (kappa = 0.72). DRS detected high-risk sites with 92% sensitivity and 60% specificity and differentiated high-risk sites from inflammation with a sensitivity and specificity of 79%. When DRS was used to target biopsies during endoscopy, the number of low-risk biopsies taken would decrease by 60%, which could save significant endoscopist and pathologist time with consequent financial savings. A negative spectroscopy result would exclude high-grade dysplasia or cancer with an accuracy of 99.5%.

Like a variant of ESS, low coherence enhanced backscattering (LEBS) spectroscopy was recently proposed as an endoscopic prescreening method for colorectal cancer screening. Based on the concept of field carcinogenesis, micro-scale spectral changes were detectable and were measured in biopsy specimens of the endoscopically normal rectum. Two hundred and nineteen patients took part in colonoscopy to predict if their colon harbored malignancy or advanced adenomas elsewhere. Alterations in the endoscopically normal rectum were seen with the help of LEBS to mirror neoplasia progression in patients with no neoplasia, 5-mm to 9-mm adenomas and advanced adenomas. For advanced adenomas, the signals from LEBS had a sensitivity of 100% with a specificity of 80% [135]. Further works with a fiber-optic probe compatible with conventional endoscopy are underway for validation of these results in vivo [129].

### Light-scattering spectroscopy

Detecting singly scattered photons, rather than diffused photons from tissue, light-scattering spectroscopy (LSS) obtains quantitative information of nuclear morphology by extracting the singly backscattered light from epithelial layers of human tissue using modeling. Due to the small optical-path length of singly scattered photons in tissue, variations in the spectrum related to the concentrations of absorbers and scatterers in the tissue are minimized [136]. When the light propagates in tissue, multiple scattering randomizes information about the scatterers over the effective scattering length [126]. Thus, the spectrum of light-scattering of tissues in vivo consists of three main components: a Rayleigh component due to elastic scattering by small organelles, a broad background from submucosal tissue [137], and a relatively small backscattered component due to epithelial cell nuclei. It was reported that the submucosal background could be removed by one of the polarization [136, 138–141] or spatial [142] gating techniques.

The smaller organelles have a very different scattering spectral dependence than that of the nuclei. The combination of gating and difference in spectral behavior allows the epithelial nuclear scattering spectrum to be isolated in the processed LSS signal [142]. The shape of the backscattered spectrum over the wavelength range is a measure of nuclear size [143], and its amplitude is related to the number density of epithelial nuclei (nuclei per unit area), which is a measure of nuclear crowding [140].

**Fig. 9:**
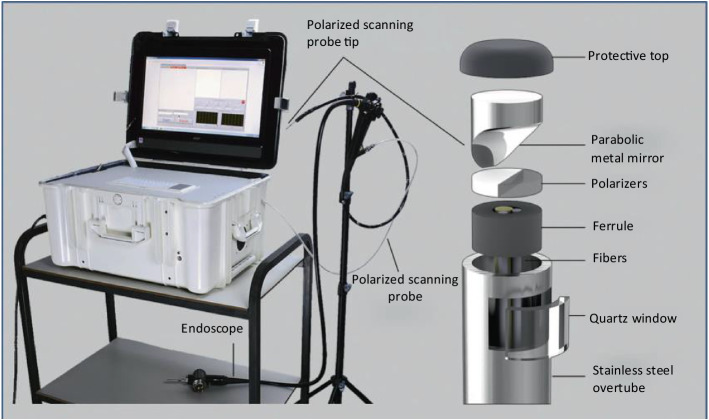
Endoscopic multispectral scanning system. The photograph on the left shows the system on a cart with its scanning probe inserted into the working channel of an Olympus GIF-H180 endoscope. The schematic on the right shows the exploded view of the polarized scanning probe tip. When assembled, the parabolic mirror is opposite the quartz window. Reproduced from Ref. [126]. Copyright 2018 Springer Nature Publishing

In 2018, Zhang et al. [142] developed an endoscopic LSS-based fiber-optic probe that predicts the malignant potential of pancreatic cystic lesions during routine diagnostic EUS-FNA procedures. In a double-blind prospective study in 25 patients, 14 cysts were measured in vivo and 13 postoperatively. The technique achieved an overall accuracy of 95%, with a 95% confidence interval of 78–99%, in cysts with a definitive diagnosis. Qiu et al. [126] proposed a multispectral probe-based detecting system that combines light scattering spectroscopy, which analyzed light scattered from epithelial cells, thereby identifying otherwise invisible dysplastic sites [138]. A compatible fiber optic probe delivered a collimated broadband light beam through the working channel (Fig. 9). The system scanned the entire Barrett's esophagus segment in minutes, providing the endoscopist with real-time operator-independent information about the location of invisible high-grade dysplasia (HGD).

They developed a rapid semi-empirical algorithm to reconstruct the epithelial cell nuclei size distribution at the illuminated spot accurately. In their algorithm, a diagnostic parameter, Δ, was provided as a measure of the contribution from dysplastic cells. The nuclear size distributions were reconstructed from the LSS data for various spatial locations. From these distributions, they determined that a site should be considered dysplastic if the diagnostic parameter exceeds 0.1. This Δ = 0.1 threshold was equivalent to ~ 25% contribution from enlarged nuclei, defined as nuclei over 9 µm in diameter.

Based on the nuclear size distributions extracted from the backscattering spectra for each individual spatial location, they made pseudo-color maps where red and pink regions indicated areas suspicious for dysplasia (Δ below 0.05 colored dark green, 0.05–0.10 colored light green, 0.10–0.15 colored pink and above 0.15 as red).

**Fig. 10:**
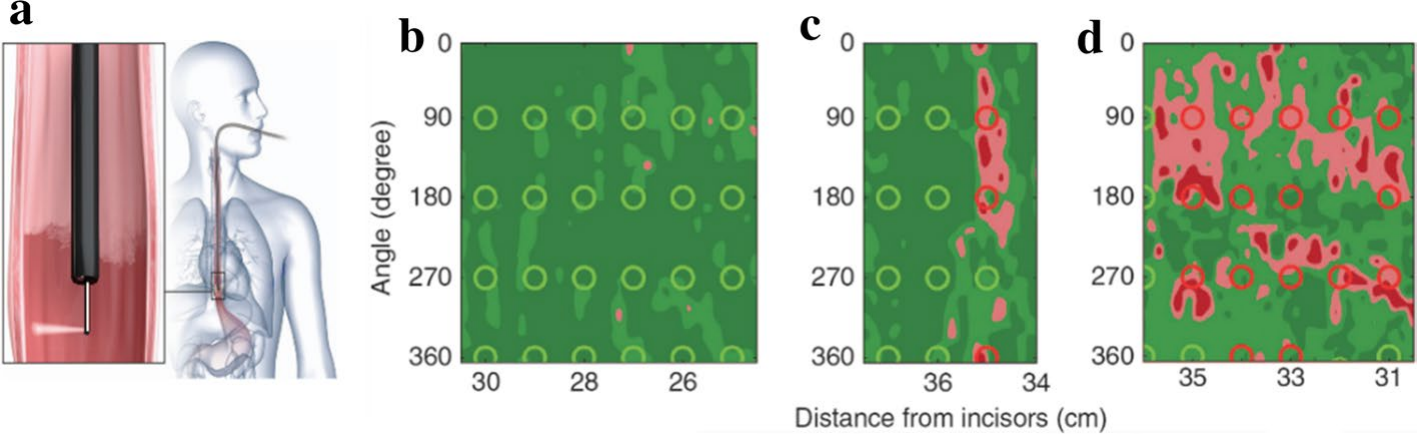
Schematic diagram of multispectral-LSS system. **a **The multispectral-LSS fiber probe performs rapid automated rotational/longitudinal scanning of the entire Barrett’s esophagus segment during the clinical procedure. **b**–**d** Pseudo-color maps produced from LSS data are overlaid with circles indicating biopsy sites and confirmed pathology. The vertical direction indicates the angle of rotation from the start of each rotary scan; the horizontal direction shows the distance from upper incisors. Green map areas of various shades represent epithelium unlikely for HGD, and red and pink map areas represent areas suspicious for HGD, as determined by LSS. Red and green circles indicate biopsy sites of HGD and non-dysplastic Barrett’s esophagus, respectively, as determined by pathology. Typical maps and biopsies for subjects with **b** no areas suspicious for HGD, **c** focal HGD suspicious areas, and **d** significant HGD suspicious regions, respectively. Adapted with permission from Ref. [126]. Copyright 2018 Springer Nature Publishing

In this way, the maps were displayed and presented to the endoscopists in cases where the multispectral endoscopic system was employed to guide biopsy (Fig. 10). In the per-patient evaluation study, the multispectral light-scattering endoscopic system demonstrated a sensitivity of 96% and a specificity of 97% for high-grade dysplasia detection. Moreover, in the per-biopsy evaluation study, a specificity of 91%, a sensitivity of 88%, and a negative predictive value of 96% in detecting individual locations of HGD were demonstrated, which reached the PIVI thresholds established by American Society for Gastrointestinal Endoscopy (ASGE) Technology Committee [144].

### Angle-resolved low coherence interferometry

Angle-resolved low coherence interferometry (a/LCI) is a novel scattering technique that combines the capabilities of light-scattering spectroscopy to detect morphological changes in cell nuclei with the depth-resolving power of optical coherence tomography. It obtains morphological information from subsurface sites by examining the depth-resolved angular distribution of elastically backscattered light [145]. A/LCI utilizes broadband light to separate light that is scattered from varied depths in tissue by mixing scattered light with that from a reference beam. By combining the low coherence interferometry with the capacity of light scattering methods, it can get structural information with subwavelength precision and accuracy [146]. Angular distribution of scattered light was also collected, then it was compared to various angular scattering solutions from a performed database to determine the average nuclear size and index of refraction from the scatters in question. There are numerous exciting applications of this technology.

Initially validated in a study measuring the size of polystyrene microspheres suspended in a neutrally buoyant medium [147], the a/LCI was also applied to measure cellular morphology in vitro [146]. Then, the a/LCI system was reported to assist in earlier detection of tissue dysplasia, resulting in more effective medical intervention and improved patient outcomes: Zhu et al. [148] proposed a probe-based endoscopic Fourier-domain a/LCI system to investigate the presence of dysplasia in vivo in Barrett’s esophagus patients. Unique nuclear size and density information were obtained from each depth layer of the tissue, which indicated the ability of a/LCI system to retrieve depth-resolved in vivo morphological and optical information from the tissue in question. In addition, the fiber probe featured long length, small diameter, and subsecond data acquisition (0.4 s/frame). Obtained depth-resolved sizing patterns were closely associated with the pathological tissue conditions and can serve as a basis for potential assessment of tissue health. Moreover, Terry et al. [149] reported sensitivity as high as 100% and specificity of 84% for the discrimination of dysplasia with a/LCI fiber probe system in 46 patients who were undergoing routine surveillance in vivo for Barrett’s esophagus. In addition, Ho et al. [150] found a strong relationship between nuclear enlargement at the basal/parabasal epithelial bin for clinical detection of cervical dysplasia and the presence of dysplasia in histological analysis.

Currently, the most significant issue is determining the state of tissue health based on average cell nuclei size [151]. Data collected will serve as a training set to establish the decision lines for future prospective studies of grading biopsy sites. Thus, more advanced hybrid algorithms, and the implementation of processing software, are still waiting to realize real-time processing and interpretation of a/LCI scan results [150].

### Raman spectroscopy

**Fig. 11:**
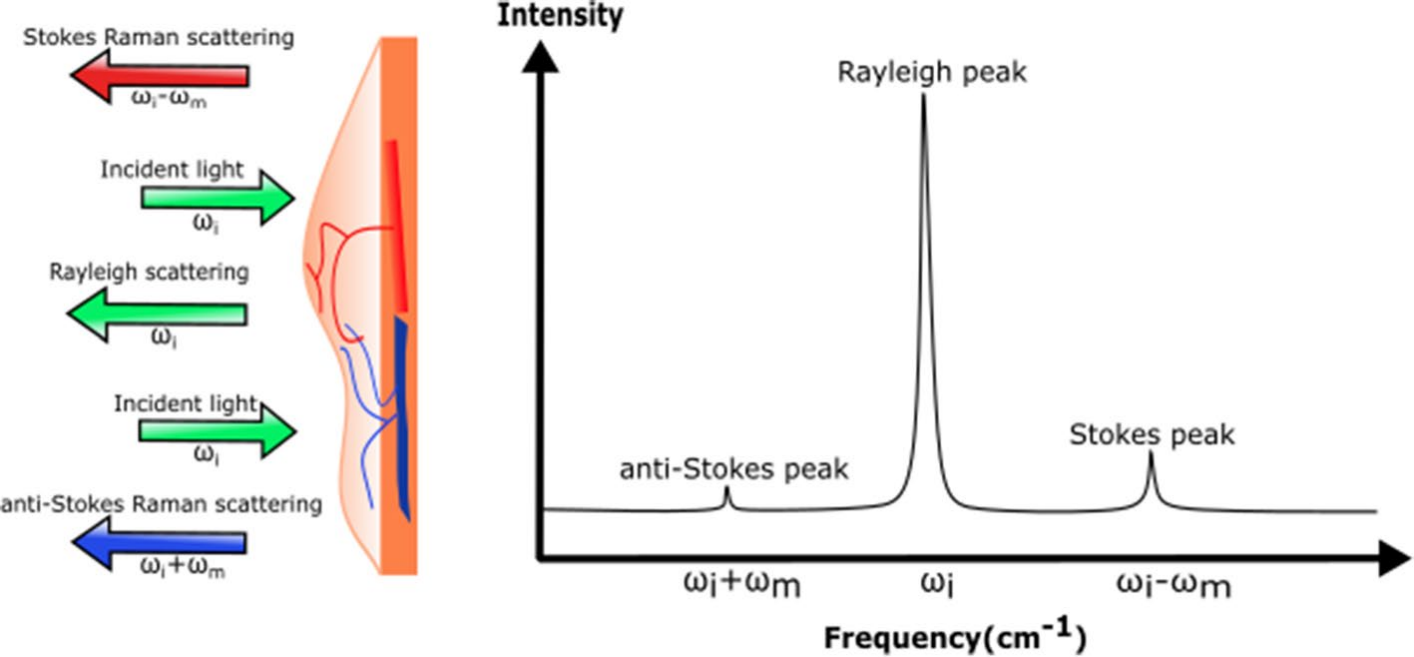
The schematic representation of Raman spectroscopy for detecting the biomolecular bonds of the target tissue. *ω*_i_ is the frequency of the incident light, while the ω_m_ is the vibrational frequency of biomolecule

Raman spectroscopy (RS) is an exciting analytical optical spectroscopic technique capable of probe vibrational and rotational modes of endogenous biomolecules of tissue or cells intrinsically. Photons in the incident laser light interact with the tissue and undergo inelastic collisions with molecules, resulting in an exchange of energy and a change in frequency. Moreover, in the Raman spectrum, the intensity of the peak of shifted light is directly proportional to the concentration of the sum of molecular constituents giving rise to that peak. Thus, the Raman spectrum is a direct function of the molecular composition of the targeted region within the tissue, giving us a complex molecular fingerprint. Most biological molecules have distinct vibrational energies in their molecular bonds. Thus, they have their specific fingerprint [152]. In addition, the Raman shift caused by frequency change is specific to the species of the molecule. The shifts in wavelengths are expressed as wave numbers, and they are independent of the wavelength of excitation, which means that the energy shift is constant for particular molecular bonds (Fig. 11).

Thus, by measuring the molecular specific inelastic scattering of light, RS enables the explanation of a tissue’s biochemical fingerprint. Primarily, RS requires a monochromatic laser to illuminate the tissue sample. Subsequently, the intensity and wavelength of the scattered light are collected and analyzed. Therefore, RS can detect subtle biochemical and molecular changes that are vital in the discrimination of tissue samples, which makes RS can become a potential diagnostic tool [153]. However, the inherent weakness of Raman signals is one major limitation of RS. Thus, endoscopic RS systems need high laser powers and ultrasensitive detectors, which are enough to merit this technique with clinical value. Moreover, the sensitivity can also be increased with the Raman signals enhanced by ten orders of magnitude by a phenomenon known as Surface Enhanced Raman Spectroscopy (SERS) [90].

Aiming to develop as a histopathology tool, Shim et al. [154] pioneeringly developed a custom-built fiber-optic Raman spectroscopic system at 785-nm excitation. Raman spectra were obtained from various organs in the digestive tract during gastrointestinal endoscopy, including the esophagus and colon, but because of the limited set of data, only subtle differences were observed in vivo between normal and pathologic states. Later on, distinctive Raman spectra were shown by the same group in a comparison between 10 adenomas and 9 hyperplastic colon polyps with 95% accuracy [155].

Furthermore, Huang et al. [156] employed a fiber-optic Raman endoscopic system for rapid Raman measurements on gastric tissue, which demonstrated for the first time that image-guided Raman endoscopy had a promising potential for the non-invasive in vivo diagnosis and detection of gastric cancer at the molecular level. In their system, wide-field images (white-light imaging/autofluorescence imaging/narrow-band imaging) and the corresponding real time in vivo Raman spectra of the tissue can be displayed and recorded simultaneously. Upon analysis of specific biochemical constituents and biomolecular differences from gastric Raman spectra, algorithms based on the Classification and Regression Tree (CART)-Raman model were developed for distinguishing neoplastic lesions from healthy tissue. The diagnostic sensitivity of 94.0% and specificity of 93.4% could be achieved.

**Fig. 12:**
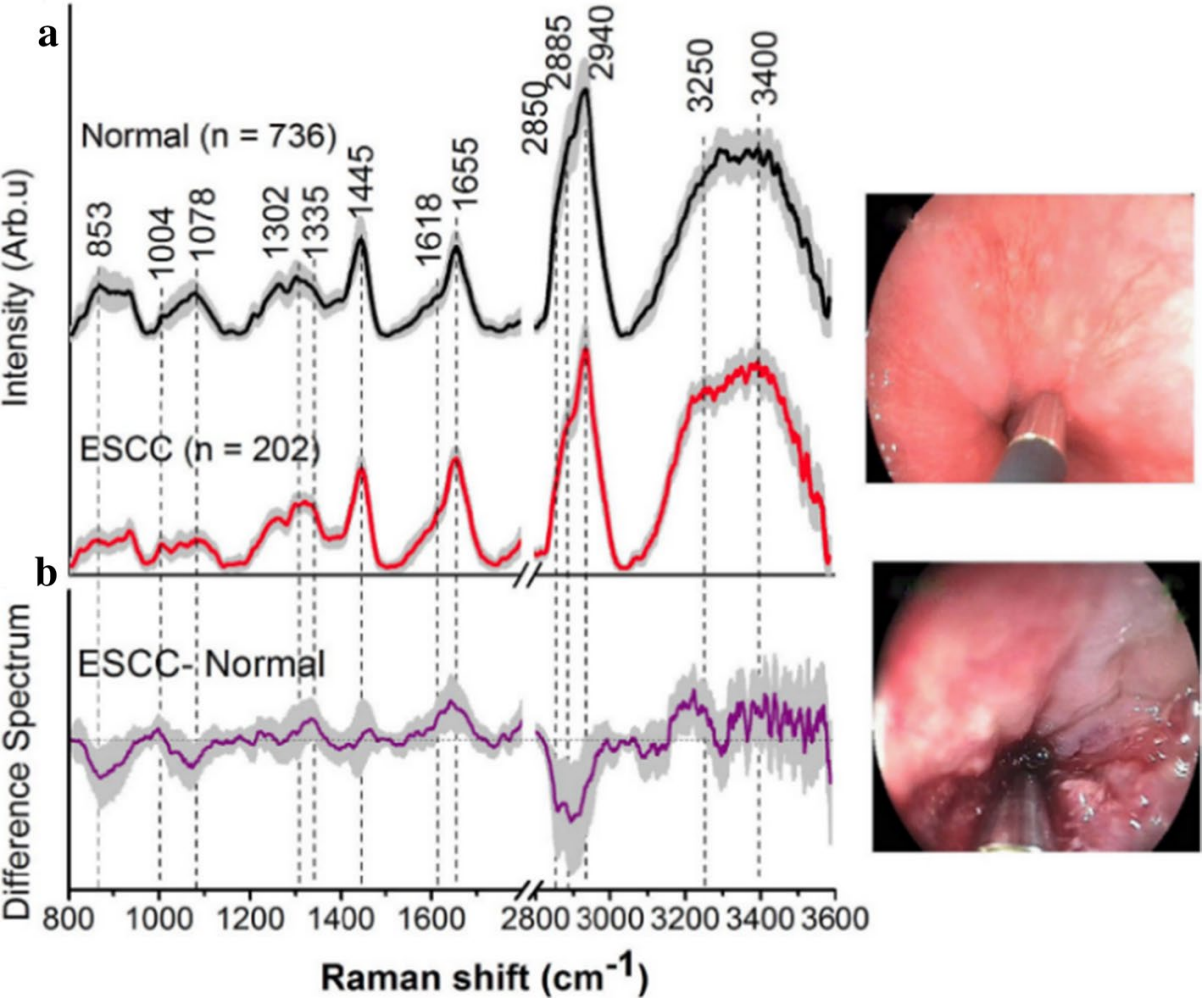
**a** The mean in vivo FP/HW Raman spectra ± 1 standard deviation (SD) of the training dataset (80% of the total dataset) (normal (*n* = 736); ESCC (*n* = 202)) for diagnostic algorithms development; **b** Difference spectra (ESCC—normal) ± 1 SD resolving the unique spectral features of ESCC. The corresponding images of the WLR-guided FP/HW Raman procedures on normal esophagus and ESCC are also shown. Adapted with permission from Ref. [157]. Copyright 2015 Springer Nature Publishing

In 2015, Wang et al. [158] developed a real-time fiber-optic Raman spectroscopy system capable of simultaneously acquiring both fingerprint (FP) (800–1800 cm^−1^) and high-wavenumber (HW) (2800–3600 cm^−1^) Raman spectra from gastric tissue in vivo. Multiple spectra for each tissue site were measured with a scanning time of 0.1–0.5 s, which permitted a rapid survey of large tissue areas. Based on the use of complementary biochemical/biomolecular information harvested through the integrated FP/HW Raman spectroscopy, the diagnosis of gastric dysplasia was enhanced as compared to either the FP or HW Raman techniques alone. Robust spectral diagnostic models were developed based on the implementation of the partial least squares discriminant analysis (PLS-DA) and leave-one-patient-out cross-validation (LOPCV). The system was finally able to provide diagnostic sensitivities of 96.0%, 81.8%, and 88.2%, and specificities of 86.7%, 95.3%, and 95.6%, respectively, for the classification of normal, dysplastic and cancerous gastric tissue, which was superior to WLE or FP or HW Raman techniques alone. Furthermore, the same group also demonstrated that their device could also improve real time in vivo diagnosis of esophageal squamous cell carcinoma (ESCC) during endoscopy [157]. In their clinical trial, a total of 1172 in vivo FP/HW Raman spectra were acquired from 48 esophageal patients. They split the total Raman dataset into two parts: 80% for training, 20% for testing (Fig. 12). The result showed that simultaneous FP/HW Raman spectroscopy on training dataset provided a diagnostic sensitivity of 97.0% and specificity of 97.4% for ESCC classification. The diagnostic algorithm applied to the independent testing dataset based on simultaneous FP/HW Raman technique gave a predictive diagnostic sensitivity of 92.7% and 93.6% specificity for ESCC identification, which is superior to either FP or HW Raman technique alone.

As far as we know, RS has excelled in the early detection of precancer and cancer in the interior of various organs with high diagnostic specificity (Table 3) [158–163]. Several probes are currently undergoing construction, and engineering tolerance levels are being carefully defined before a clinical trial [164]. Further evaluations of the clinical merits of Raman spectroscopy for prospective prediction of precancer and cancer in vivo are expected.

**Table 3:** In vivo human clinical trial researches on novel endoscopic optical diagnostic techniques in primary medical specialities

Technique	References									
	Gastroenterology					Urology	Gynecology	Pneumology	Otorhinolaryngology	Laparoscopic surgery
	Pancreatic duct	Biliary tract	Upper GI tract	Lower GI tract						
HSI	–	–	[30, 165, 166]	[23]		–	[167]	–	[29, 168]	–
FLIM	-	–	[46, 49]	[169]		[170]	-	–	–	–
PAE		–	–	-		–	–	–	–	
OCT	[171]	[172, 173]	[94, 96, 174, 175]	[91, 176, 177]		[95, 178, 179]	[93, 180, 181]	[174, 182, 183]	[184, 185]	[186]
CASR	–	–	–	–		–	–	–	–	–
MPE	–	–	–	–		–	-	–	–	–
DRS	–	–	[187]	[188]		[189]	[190–192]	-	–	–
LSS	[142]	–	[190, 193, 194]	[124, 195]		[195]	[190]	–	–	–
a/LCI	–	–	[148, 149]	–		–	[150]	–	–	–
RS	–	–	[158, 161, 162, 196]	[163, 197]		[198]	[199–201]	–	[202–204]	–

## Discussion

Before any novel endoscopic optical diagnostic technology is successfully put into clinical use, it is necessary to conduct ex vivo/in vivo preclinical animal experiments and ex vivo human trials, which are followed by rigorous in vivo human clinical trials to further verify the effectiveness and safety of these techniques. We track the emerging in vivo human clinical trial researches on novel endoscopic optical diagnostic techniques and summarize their coverage on several primary medical specialties in Table 3, which also indicates unexplored speciality fields for clinical researchers. In addition, the challenges, as well as the future development orientation of endoscopic optical diagnostic techniques, are discussed below.

### Future perspectives

**Fig. 13:**
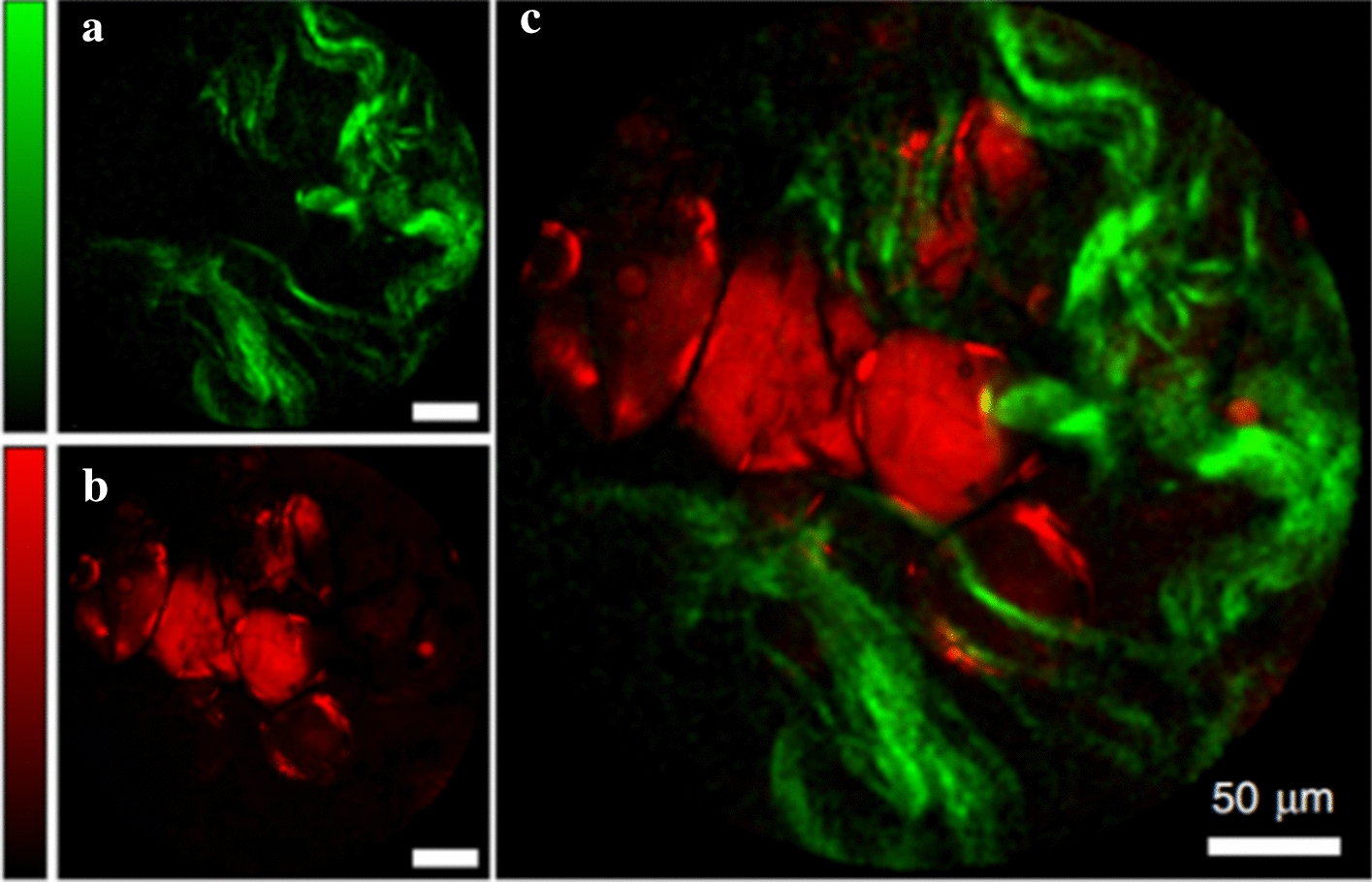
Multimodal SHG (green, **a**) and CARS (red, **b**) images of fresh human colon tissues. Images were taken 50 μm below the sample surface. **c** The large images are overlaps of the SHG and CARS images. Adapted with permission from Ref. [205]. Copyright 2018 Springer Nature Publishing

Future work related to the advancements of endoscopic imaging and sensing may focus on:

1. A higher resolution with a wider field of view.
2. An increased frame rate with super-resolution.
3. Combining multiple modalities. Compared to one technique, several characteristic information can be obtained simultaneously, which cannot be acquired by these technologies individually. For instance, Lombardini et al. [205] combined CARS and SHG during endoscopy, which acquire SHG images immediately after the CARS images (Fig. 13).
4. Improving the image signal-to-noise ratio to highlight lesion characteristics.
5. Miniaturization of the probes. We prospect that further miniaturization of the flexible probes will benefit diagnosis by covering more specific medical scenarios. The birth of new materials has driven the miniaturization of detectors. For instance, in 2019, Yang et al. [206] developed miniaturized spectrometers based on the single, compositionally engineered nanowire. Their devices are capable of accurate, visible-range monochromatic and broadband light reconstruction, as well as spectral imaging from centimeter-scale focal planes down to lensless, single-cell-scale in situ mapping. In this way, the single-nanowire spectrometers can detect the spectral information of biological tissue in hyperspectral imaging.
6. Furthermore, we envision that future works on the advance of endoscopic modalities could shed light on transferring bench-top super-resolution microscopies (SRM) into miniature and flexible imaging devices such as endoscope-based probes or catheters. In this way, they could provide new mechanistic insights into biological processes.

**Fig. 14:**
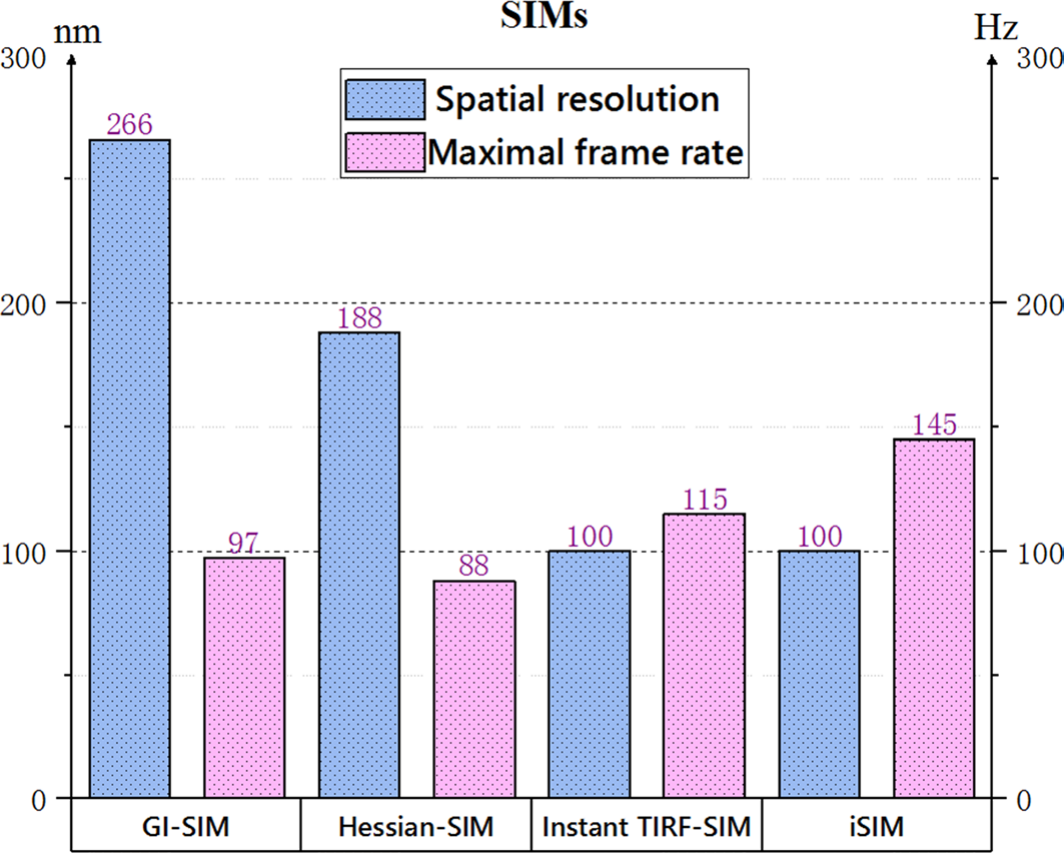
The spatiotemporal resolution of various structured illumination microscopies. GI-SIM, SIM with grazing incidence illumination [219, 224], Hessian-SIM, Hessian structured illumination microscopy [218]; instant TIRF-SIM, instant total internal reflection fluorescence structured illumination microscopy [221]; iSIM, instant structured illumination microscopy [220]

The imaging resolution of SRM breaks the Abbe limit and is above 200 nm. Owning sub-micron or even nano-scale optical discrimination ability, SRM like photoactivated localization microscopy (PALM) [207–209], stochastic optical reconstruction microscopy (STORM) [210–212], stimulated emission depletion (STED) [213–216] and structured illumination microscopy (SIM) [217–224] can continuously monitor the evolution of macromolecules and organelles without affecting the biological activity of biological systems. Among various SRMs, SIM excelled in extending the spatiotemporal resolution and the imaging duration with a notably reduced illumination intensity. Moreover, SIMs are now the most widely used technique in super-resolution optical microscopy of living cells [225]. Thus, SRMs are vital tools to research life sciences. Figure 14 illustrates the spatiotemporal resolution of these SIM systems.

### Challenges

**Fig. 15:**
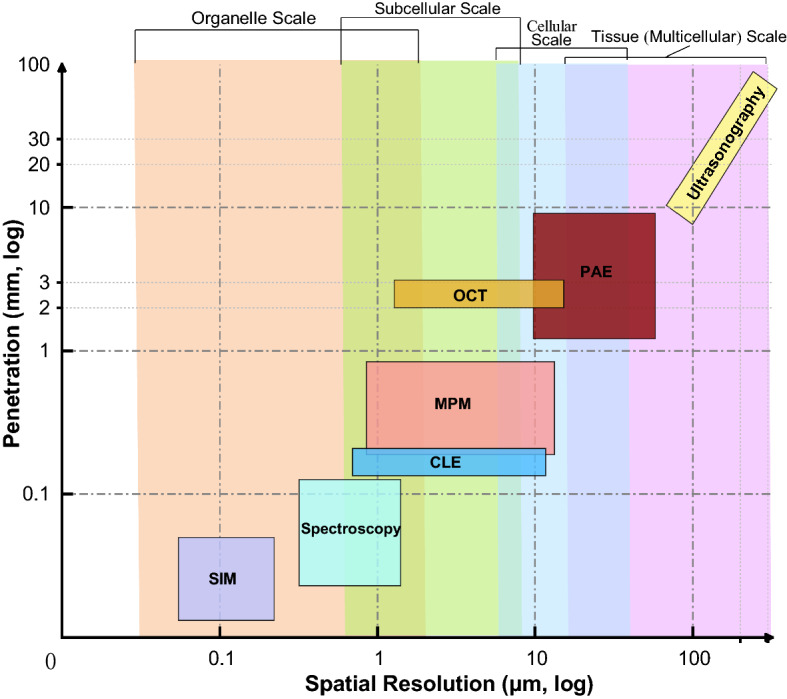
Multiple techniques used for tissue diagnosis with varying spatial resolution and penetration depths. The four colored regions in the background represent different biological scales: from the organelle to tissue (multicellular) scale

1. As we know, the targeted objects detected by clinical endoscopy are all three-dimensional. Both surface and depth information are used to diagnose the disease. However, there is a significant barrier to quantitative characterization of the targeted tissue: imaging towards biomedical utilization has an inverse relationship between the volume that any imaging modality can cover (field of view and penetration depth) and the size of details it can visualize (spatial resolution) [226, 227]. Thus, emerged imaging modalities are often restricted to a single range for the combined resolution, field of view, and penetration depth [228]. In practical terms, those imaging techniques utilized in endoscopy usually acquire information on among organelle scale, subcellular, cellular scale, and tissue (multicellular) scale (Fig. 15). As for a particular modality, the higher spatial resolution is achieved, the deeper detectable depth it abandons.
2. The real-time performance of an endoscopic imaging system is a crucial indicator accounting for diagnostic efficiency. However, temporal resolution is limited by scanning speed, sample fluorescence signal intensity, detector sensitivity, and processing time. Several technologies such as THG, CARS, and PAE are not yet real time, with frame rates below 5 Hz, thereby increasing the patient's suffering during clinical procedures.
3. The diagnostic capability of “optical biopsies” has not reached that of ex vivo biopsy. Because “optical biopsies” has some limitations: (i) it does not reach the detection depth as fine-needle aspiration (FNA); (ii) can not observe cells cultured in vitro for subsequential changes. Also, the optical biopsy result consistency should be evaluated because different endoscopists could lead to variable diagnostic results. Thus, the uniform standards of image data interpretation and technical specifications should be set clearly before clinical practice.
4. Another limitation is that it is difficult to judge whether the light projected on the tissue is safe. Thus, the power, wavelength, and duration of the light should be considered and calculated. In addition, studies on phototoxicity are required to identify the most discriminating wavelengths and lifetimes to create a relevant diagnostic model [129].
5. Meanwhile, there sorely lacks software tools for interpreting clinical data accurately and the guidance for the use of software for the different observers.
6. Furthermore, it is essential to develop endurable systems that are also easy to be sterilized.
7. Lastly, ethical acceptability needs to be considered and evaluated.

## Conclusion

This review aims to help researchers to have a broad and clear understanding of emerging innovative endoscopic diagnostic technologies in medical trial researches, we mainly summarize studies on these promising techniques, with emphasis on their development processes and recent advances. We also contribute to cover both the basic principles and technical parameters of these technologies. We exploit the innovative optical imaging and sensing mechanisms of these prototype technologies, which promoted an unprecedented stride in basic scientific and clinical trial studies. We further explain how these technologies aids endoscopists to perform accurate and time-efficient optical diagnosis with real-time functional and spatial structural information on regions of interest.

Among all novel techniques in medical trial research we stated above, endoscopic OCT is relatively mature by now. A large number of successful clinical experiments have been conducted on different endoscopic OCT systems to validate their application in many medical specialties. Similar to OCT, PAE can also construct 3D stereo images in vivo with cellular resolution and millimeter-scale penetration depth. HSI acquires morphologic and physiologic information and provides a 3D “data tube” comprised of 2D spatial and 1D spectral details. Furthermore, FLIM differentiates carcinoma from healthy tissue by recording the fluorescence lifetimes which are recorded by each pixel in a particular field of view. Moreover, for prescreening, DRS, LSS, and a/LCI measure the average size and optical density of cell nuclei in epithelial tissue to identify potentially harboring neoplasia. RS reveals specific biomolecule fingerprint before morphologic changes by measuring the shift of wave numbers. In addition, promising endomicroscopic technologies such as MPE and CARS provide real-time subcellular imaging, but they have not been reported in any in vivo clinical experiments in any medical specialty (as shown in Table 3).

Furthermore, these emerging novel endoscopic optical diagnostic techniques will continue to develop with the cooperation of multi-disciplinary to improve the diagnostic capabilities of detection, characterization, and confirmation in the near future. To obtain certification of the supervisory authorities on medical devices in different regions all over the world, such as Food and Drug Administration (FDA) and European Conformity (CE), current efforts are directed to implement and validate these technologies for modern endoscopy before general clinical practice in hospitals. However, these emerging diagnostic devices have not obtained official approval of clinical use and vary across maturities. Some challenges lie in the results inconsistency, cost-effectiveness, time-efficiency, etc., which require further researches and developments to achieve a promising era of endoscopy.

## Data Availability

Not applicable.

## Abbreviations

NBI: Narrow-band imaging
LCI: Linked color imaging
CLE: Confocal laser endomicroscopy
HSI: Hyperspectral imaging
FLIM: Fluorescence lifetime imaging microscopy
CLE: Confocal laser endomicroscopy
OCT: Optical coherent tomography
PAE: Photoacoustic endoscopy
DRS: Diffuse reflectance spectroscopy
LSS: Light-scattering spectroscopy
a/LCI: Angle-resolved low coherence interferometry
RS: Raman spectroscopy
CARS: Coherent anti-Stokes Raman scattering
TPEF: Two-photon excitation fluorescence
3PEF: Three-photon excitation fluorescence
SHG: Second-harmonic generations
THG: Third-harmonic generations
SRM: Super-resolution microscopy
SIM: Structured illumination microscopy
PALM: Photoactivated localization microscopy
STORM: Stochastic optical reconstruction microscopy
STED: Stimulated emission depletion
